# A genome-wide CRISPR screen maps endogenous regulators of PPARG gene expression in bladder cancer

**DOI:** 10.1016/j.isci.2023.106525

**Published:** 2023-03-30

**Authors:** Davide Tortora, Morgan E. Roberts, Gunjan Kumar, Sudha S. Kotapalli, Elie Ritch, Joshua M. Scurll, Brian McConeghy, Sunita Sinha, Alexander W. Wyatt, Peter C. Black, Mads Daugaard

**Affiliations:** 1 Department of Urologic Sciences, Vancouver Prostate Centre, University of British Columbia, Vancouver, BC V6H 3Z6, Canada; 2 Sequencing and Bioinformatics Consortium, University of British Columbia, Vancouver, BC V6T 1Z3, Canada

**Keywords:** Biological sciences, Molecular biology, Molecular mechanism of gene regulation, Cancer

## Abstract

Peroxisome proliferator-activated receptor gamma (PPARγ) is a nuclear receptor central in the regulation of key cellular processes including cell metabolism, tissue differentiation, and regulation of the immune system. PPARγ is required for normal differentiation of the urothelium and is thought to be an essential driver of the luminal subtype of bladder cancer. However, the molecular components that regulate *PPARG* gene expression in bladder cancer remain unclear. Here, we developed an endogenous *PPARG* reporter system in luminal bladder cancer cells and performed genome-wide CRISPR knockout screening to identify *bona fide* regulators of *PPARG* gene expression. Functional validation of the dataset confirmed GATA3, SPT6, and the cohesin complex components SMC1A, and RAD21, as permissive upstream positive regulators of *PPARG* gene expression in luminal bladder cancer. In summary, this work provides a resource and biological insights to aid our understanding of *PPARG* regulation in bladder cancer.

## Highlights

• An endogenous *PPARG* reporter system in luminal bladder cancer cells was developed
• A genome-wide CRISPR knockout screen revealed putative regulators of *PPARG* gene expression
• GATA3, SPT6, SMC1A, and RAD21 were validated as positive regulators of *PPARG*
• This work provides a resource for insight into *PPARG* regulation in bladder cancer

## Graphical abstract

**Figure:**
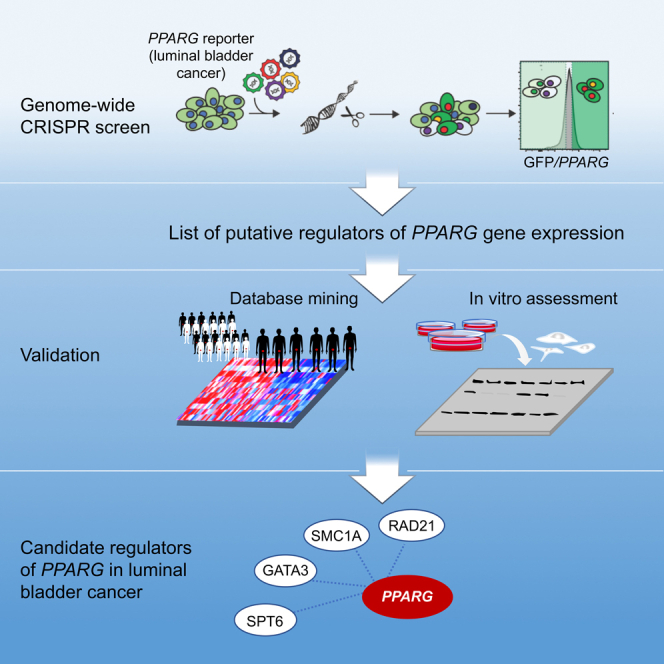

## Introduction

Peroxisome proliferator-activated receptor gamma (PPARγ) is a nuclear receptor responsible for integrating multiple biological signals and orchestrating appropriate downstream gene expression responses.^1^ It is a ligand-activated transcription factor that heterodimerizes with its obligate partner, retinoid X receptor α (RXRα), and binds DNA on a motif known as a peroxisome proliferator response element.^2^^,^^3^^,^^4^ PPARγ has been widely studied for its essential roles in numerous biological mechanisms including adipocyte differentiation, lipid metabolism, insulin sensitization, and immunity.^5^^,^^6^ Furthermore, it has also been implicated as a tumor-promoting factor in several cancer types, including breast, colon, prostate, and bladder cancer.^7^^,^^8^^,^^9^^,^^10^^,^^11^^,^^12^^,^^13^ However, the role of PPARγ in cancer is somewhat controversial as it was initially thought to have anti-neoplastic effects.^14^ The biology of PPARγ activity is complex and highly context dependent,^6^^,^^15^ which likely underlies much of the disparity between the various studies.

PPARγ is now well defined as a key transcription factor in a major subset of bladder cancer, in which it acts to promote tumor growth through cell intrinsic and extrinsic mechanisms.^9^^,^^13^^,^^16^^,^^17^^,^^18^^,^^19^^,^^20^ Bladder cancer is the 10^th^ most common malignancy worldwide, has a high rate of morbidity and mortality, and is a significant burden on global healthcare systems.^21^^,^^22^ Recent work has shown that bladder cancer can be segregated into luminal and basal subtypes based on gene expression profiles.^17^^,^^18^^,^^23^^,^^24^ The majority of bladder cancer is characterized as luminal, which makes up approximately 91%–94% of non-muscle invasive bladder cancer and 47% of muscle invasive bladder cancer (MIBC).^23^^,^^24^ PPARγ activity has been identified as a key feature of luminal tumors,^9^^,^^17^^,^^18^^,^^25^ and PPARγ has been shown to be oncogenic in PPARγ hyperactive tumors.^9^^,^^10^ Approximately 40% of MIBC tumors exhibit high PPARγ activity, which results from activating mutations in *RXRA*, copy number amplification of *PPARG*, or increased *PPARG* gene expression.^16^^,^^26^ Of these mechanisms, upregulation of *PPARG* mRNA levels is the most common, with 27% of MIBC tumors having high *PPARG* expression in the absence of copy number alterations.^16^ However, the molecular mechanisms that regulate *PPARG* expression in luminal bladder cancer remain unclear.

At the crossroads of multiple molecular pathways, PPARγ is central in the regulation of cellular metabolism, tissue differentiation, and immune responses.^5^^,^^27^^,^^28^ These aspects are fundamental to cancer development and progression, and thus, unraveling the regulatory mechanisms that control *PPARG* gene expression in bladder cancer has important implications for the understanding and treatment of the disease. Furthermore, as PPARγ is a regulator of normal urothelial differentiation,^29^^,^^30^ knowledge of its regulation is important for understanding the fundamental biology of bladder development and function. We performed a genome-wide CRISPR screen to identity endogenous regulators of *PPARG* gene expression in bladder cancer. Our data provide insight into the molecular mechanisms regulating *PPARG* gene expression and identify potential therapeutic targets in this pathway.

## Results

### Design and validation of a genetic reporter system to monitor endogenous *PPARG* gene expression

In order to study the regulation of *PPARG* gene expression in bladder cancer, we developed a cell-based reporter system that reflected endogenous changes in *PPARG* expression. Since high *PPARG* expression is a feature of luminal MIBC, we sought to identify a luminal bladder cancer cell line with high endogenous levels of *PPARG* to use for the assay. Using previously published RNA sequencing (RNAseq) data and molecular subtyping calls, 17 luminal bladder cancer cell lines were identified.^18^ Consistent with previous reports, many luminal cell lines were enriched for *PPARG* mRNA (Figure S1A). UM-UC1, UM-UC9, UM-UC14, and RT112 cells also had high PPARγ expression at the protein level, with UM-UC9 having the highest expression, consistent with a known amplification of the *PPARG* gene (Figure S1B).^16^^,^^31^ Based on these data, and the consistency in subtyping of RT112 cells as luminal by multiple groups and methods,^18^^,^^32^^,^^33^ RT112 cells were selected to develop our reporter system.

**Figure 1:**
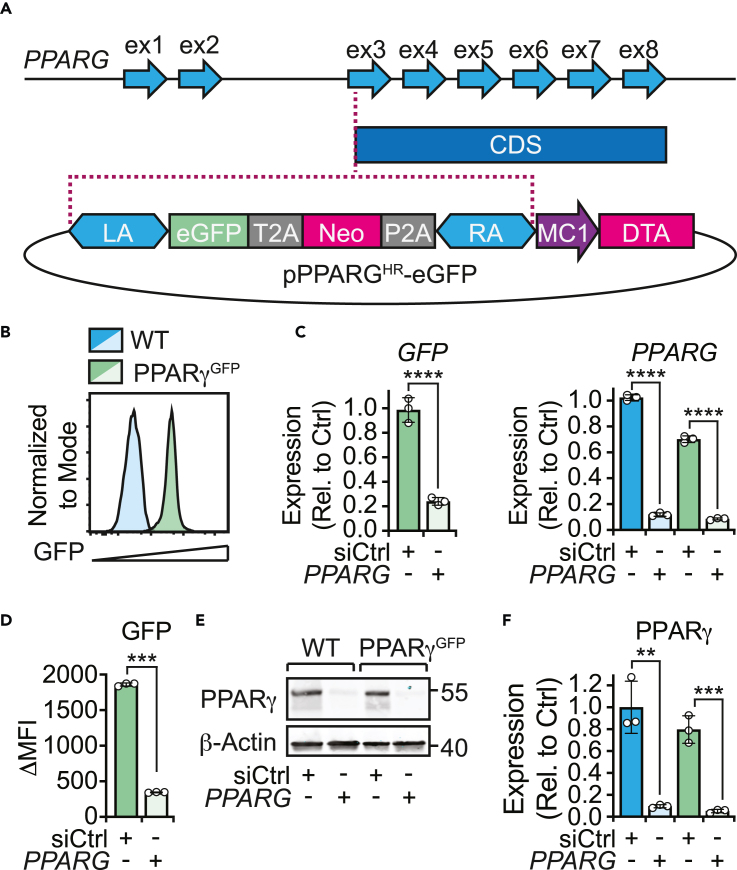
Development of a *PPARG* gene expression reporter system (A) Diagram illustrating the reporter vector (pPPARG^HR^-eGFP, 7086bp) and its intended insertion point in the genome (dotted line). Enhanced green fluorescent protein (eGFP), neomycin resistance (Neo), peptide self-cleaving sequences (T2A, P2A), homology arms (LA, RA), MC1 promoter, diphtheria toxin (DTA), and *PPARG* features (coding sequence (CDS), exons (ex1-8)) are highlighted. (B) Histogram represents green fluorescence (eGFP) of wild type (WT, blue) and PPARγ^GFP^ (green) RT112 cells. (C–F) *PPARG* was knocked down in WT and PPARγ^GFP^ RT112 cells by siRNA. (C) mRNA expression for the indicated genes was assessed by RT-qPCR. Graphs represent gene expression relative to matched siCtrl samples. (D) Expression of eGFP was assessed by flow cytometry. Graph represents delta median fluorescence intensity (ΔMFI) relative to WT control cells. (E and F) PPARγ protein levels were assessed by Western blot and β-actin was used as a loading control. Numbers indicate molecular weight (kDa) of closest molecular weight marker. (F) Graph represents relative intensity of the band compared with the corresponding siCtrl sample. Each sample was first normalized relative to its corresponding β-actin band. (C, D, and F) Data plotted as mean (n = 3) with standard error of mean (SEM). Statistical significance as determined by student’s t-test. ∗p < 0.05, ∗∗p < 0.01, ∗∗∗p < 0.001, ∗∗∗∗p < 0.0001.

To generate a reporter system able to monitor variations in endogenous *PPARG* gene expression, enhanced green fluorescence protein (eGFP) and neomycin resistance genes were inserted immediately upstream of, and in frame with, the *PPARG* coding sequence in RT112 cells (Figure 1A). Given the size of eGFP and its propensity for dimerization,^34^ a fusion of eGFP to PPARγ would likely affect the functionality of PPARγ. Therefore, 2A sequences, encoding self-cleaving peptides, were introduced following the eGFP and neomycin resistance genes. This created a fusion gene under the transcriptional control of the endogenous *PPARG* regulatory system, without affecting the downstream function of PPARγ.

To create a double-strand DNA cut at the *PPARG* 5′ region, a plasmid encoding Cas9 and a targeting CRISPR guide RNA was transiently co-transfected with the donor plasmid (PPARG^HR^-eGFP, Figure 1A) into RT112 cells. The reporter sequence was positioned in between two 1 kb DNA sequences (left arm (LA), right arm (RA)) homologous to the regions surrounding the CRISPR cut site. This provided the cells with the appropriate substrate to fix the CRISPR-induced damage using their natural homology-directed repair (HDR) system, thus resulting in the introduction of the reporter gene at the desired locus. To increase the specificity of the HDR process, a diphtheria toxin A (DTA) cassette was added downstream of the RA sequence in the PPARG^HR^-eGFP plasmid, in order to select against random integration of the vector in non-specific regions of the genome.^35^ Moreover, to prevent DTA toxicity by its expression from the donor plasmid, and to increase the recombination efficiency,^36^ PPARG^HR^-eGFP was linearized prior to transfection. Following transfections, cells carrying the reporter construct were selected by florescence-activated cell sorting to generate a pure population of reporter cells (RT112-PPARγ^GFP^) (Figure 1B).

We next sought to validate the reporter system. First, the correct insertion of the reporter gene was confirmed by PCR amplification of sequences spanning the genomic DNA and inserted donor template (Figure S2). Next, we knocked down *PPARG* with short interfering RNA (siRNA) and evaluated changes in eGFP expression. Knockdown (KD) of *PPARG* in RT112-PPARγ^GFP^ cells led to a 76% and 87% decrease in *GFP* and *PPARG* mRNA levels, respectively, compared to scrambled siRNA controls (siCtrl) (Figure 1C). This was consistent with an 81% decrease in GFP fluorescence and a 93% decrease in PPARγ protein (Figures 1D–1F). We then questioned whether insertion of the reporter construct affected baseline expression of PPARγ. PPARγ mRNA and protein levels were assessed in wild-type RT112 (WT) and RT112-PPARγ^GFP^ cells by RT-qPCR and Western blot. RT112-PPARγ^GFP^ cells had a 30% decrease in *PPARG* mRNA, and a 20% in PPARγ protein compared with WT cells (Figures 1E and 1F). Finally, no differences were observed in the magnitude of the *PPARG* KD between WT and RT112-PPARγ^GFP^ cells at either an mRNA (both 88%) or protein (90% and 93%, respectively) level (Figures 1C and 1F).

### Whole-genome CRISPR knockout screening reveals endogenous regulators of *PPARG* gene expression

**Figure 2:**
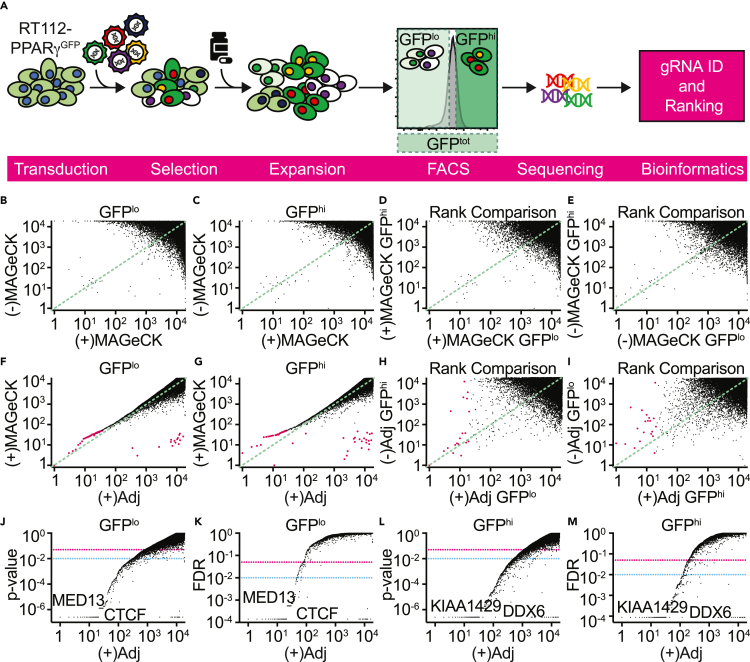
Performing the genome-wide knockout screen and ranking the hits (A) Diagram illustrating the steps of the screen. RT112-PPARγ^GFP^ cells were transduced with a CRISPR lentiviral library and transduced cells were enriched by antibiotic selection. Knockout of genes affecting *PPARG* expression resulted in altered GFP production. Live (propidium iodide negative) cells were sorted by fluorescence activated cell sorting (FACS) according to their GFP fluorescence intensity (GFP^lo^ and GFP^hi^ by approximate quartiles, and total GFP^+^ (GFP^tot^)), and sgRNA enrichment was evaluated by next-generation sequencing (NGS). (B–E) Comparison of positive (pos) or negative (neg) gene enrichment as ranked by the MAGeCK algorithm, within the (B) GFP^lo^ or (C) GFP^hi^ samples or (D-E) between samples. Genes with equivalent rank in both lists appear closer to a line of slope 1 (green dashed line). (F and G) Adjusted (Adj) ranks are compared with the corresponding MAGeCK ranks for (F) GFP^lo^ and (G) GFP^hi^ cells. Pink dots indicate top 50 hits in the positive MAGeCK rank. (H) Rank comparison between positive adjusted ((+)Adj) GFP^lo^ vs. negative adjusted ((-)Adj) GFP^hi^. Pink dots indicate top 20 hits in GFP^lo^ rank. (I) Rank comparison between positive adjusted GFP^hi^ vs. negative adjusted GFP^lo^. Pink dots indicate top 20 hits for GFP^hi^ rank. (H-I) Hits with equivalent rank in both the lists in analysis appeared closer to a line of slope 1 (green dashed line, line of rank congruency). (J–M) Distribution of p-values and false discovery rates (FDR) for hits in the (J-K) GFP^lo^ or (L-M) GFP^hi^ groups. Hits at which a drop of significance occurs are indicated. Pink dotted line = 0.05. Blue dotted line = 0.01.

To identify transcriptional regulators of steady-state PPARγ expression in the context of luminal bladder cancer, two independent whole-genome CRISPR knockout screens were performed with the RT112-PPARγ^GFP^ reporter cell line, using changes in eGFP as readout (Figure 2A). A pooled library, co-expressing Cas9 and single-guide RNAs (sgRNA) designed to target most genes in the genome (19,114 genes, four unique guides per gene), was delivered via lentivirus transduction to the reporter cell line at a multiplicity of infection of 0.3, in order to limit the number of plasmids received by each cell to one. The cells were then cultured under antibiotic selection for a period of 7 days to select against un-transduced cells (Figure S3A), to allow for genome editing to take place, and for the screened phenotypes to become detectable. Cells were then collected and live (propidium iodide negative) GFP^hi^ (upper quartile) and GFP^lo^ (lower quartile) cells were sorted by fluorescence-activated cell sorting. Total live GFP^+^ cells (GFP^tot^) were also sorted as a control group (Figure S3B). Deletion of factors involved in the upregulation of *PPARG* transcription should result in lower GFP expression, thus sgRNAs targeting those factors would be enriched in the GFP^lo^ group and depleted from the GFP^hi^, compared to the GFP^tot^ control, and *vice versa*.

Genomic DNA was extracted from the three populations (GFP^lo^, GFP^hi^, and GFP^tot^) and the abundance of each sgRNA was quantified by next-generation sequencing. The computational analysis of the CRISPR screen included guide counting and statistical tests for the assessment of guide and gene level significance, as well as quality control for sequencing, library representation, and the reproducibility of results from biological experimental replicates. Analysis of the sgRNA abundance for the GFP^tot^ control samples confirmed the uniform representation of sgRNAs in both screens with almost no difference between experimental replicates (AUC = 0.67075 and 0.67256) (Figure S4A). In addition, no significant differences were found between guide counts in the GFP^tot^ samples between screen 1 and 2, and only very small differences were observed between replicates for the GFP^lo^ and GFP^hi^ samples (Figures S4B and S4C).

Given the equivalence in sgRNA distribution in the GFP^tot^ samples, data from the two independent replicates were combined to improve power and confidence in hit calling. The 19,114 genes targeted by the CRISPR library were then ranked using the Model-based Analysis of Genome-wide CRISPR/Cas9 Knockout (MAGeCK) algorithm,^37^ thus obtaining a list of genes, ranked for their enrichment or depletion in each sample. Comparing different lists, genes with equivalent rank appear closer to a line of slope 1 (line of rank congruency). By comparing these ranks within each sample, we identified a subset of genes, which had statistically significant scores in both the lists for positive and negative enrichment (Figures 2B and 2C). A similar cluster was identified by an equivalent analysis comparing GFP^hi^ to GFP^lo^ cells (Figures 2D and 2E). This cross-list comparison identified a group of hits, including *MYC*, *EIF2AK4*, *ACTL6B*, *PELO*, *UFSP2*, *BAX*, and *CKS1B*, which were always found to be in the top 20 of any MAGeCK rank. Therefore, an additional heuristic filter was applied to filter out these potentially false positive hits. In each experimental group, enriched gene knockouts were organized according to an adjusted order, calculated as the difference between the MAGeCK-derived negative and positive ranks. This adjusted rank was used for our analyses, and, despite modifying the original MAGeCK ranking, was found to preserve the major order of the remaining potential true hits (Figures 2F and 2G).

An internal validation for the reliability of our screen came from the comparison of the adjusted hit ranks between the GFP^lo^ and GFP^hi^ cells. Top hits enriched in the positive rank from the GFP^lo^ group were also highly positioned in the negative rank from the GFP^hi^ sample (Figure 2H). This means that sgRNAs enriched in GFP^lo^ were depleted from GFP^hi^ sample and *vice versa*. This relationship persisted to a lesser degree when positive GFP^hi^ and negative GFP^lo^ ranks were compared (Figure 2I). FDR and p-value analyses showed that, despite remaining highly significant, there was a drop in significance between hits placed in the 20^th^ (CTCF) and 21^st^ (MED13) positions in the GFP^lo^ group (Figures 2J and 2K). This rank is also approximately where the hits started diverging from the line of rank congruency between the positive GFP^lo^ and negative GFP^hi^ rank (Figure 2H). A similar drop was observed at around position 35 for the GFP^hi^ counterpart of the screen (Figures 2L and 2M).

Lists of the top candidate positive and negative regulators (herein referred to as “top hits”) were then generated. Genes ranked within the top 50 enriched genes and with an FDR <0.01, for the GFP^lo^ (positive regulators) and GFP^hi^ (negative regulators) samples were included in these lists. 47 and 50 hits from the GFP^lo^ and GFP^hi^ samples, respectively, fit these criteria (Tables 1 and S1). Taken together, these analyses support the validity of the screen, and provided a list of potential regulators of *PPARG* in bladder cancer.

**Table 1:** List of putative positive regulators of *PPARG* gene expression

Adj rank^a^	Gene ID	Gene name
**1**	GATA3	GATA-binding protein 3
**2**	AHR	aryl hydrocarbon receptor
**3**	SMC1A	structural maintenance of chromosomes 1A
**4**	MED12	mediator complex subunit 12
**5**	PPARG	peroxisome proliferator-activated receptor gamma
**6**	CUL3	cullin 3
**7**	ARNT	aryl hydrocarbon receptor nuclear translocator
**8**	MAU2	MAU2 sister chromatid cohesion factor
**9**	RARG	retinoic acid receptor gamma
**10**	NIPBL	NIPBL, cohesin loading factor
**11**	KEAP1	kelch-like ECH-associated protein 1
**12**	DYRK1A	dual specificity tyrosine phosphorylation regulated kinase 1A
**13**	CARS1	cysteinyl-tRNA synthetase 1
**14**	CAND2	cullin associated and neddylation dissociated 2
**15**	SRSF2	serine and arginine rich splicing factor 2
**16**	RUNX1	runt-related transcription factor 1
**17**	SAE1	SUMO1 activating enzyme subunit 1
**18**	SUPT6H	Suppressor of Ty Homolog-6
**19**	MED23	mediator complex subunit 23
**20**	SMC3	structural maintenance of chromosomes 3
**21**	CTCF	CCCTC-binding factor
**22**	MED13	mediator complex subunit 13
**23**	CRTAP	cartilage-associated protein
**24**	EEF2	eukaryotic translation elongation factor 2
**25**	ZC3H4	zinc finger CCCH-type containing 4
**26**	RAD21	RAD21 cohesin complex component
**27**	TSEN2	tRNA splicing endonuclease subunit 2
**28**	DOT1L	DOT1-like histone lysine methyltransferase
**29**	PUM1	pumilio RNA-binding family member 1
**30**	VILL	villin like
**31**	GRIP2	glutamate receptor interacting protein
**32**	DNM2	dynamin 2
**33**	MED24	mediator complex subunit 24
**34**	HSPA5	heat shock protein family A (Hsp70) member 5
**35**	WDR82	WD repeat domain 82
**36**	MED16	mediator complex subunit 16
**37**	ZMYND8	zinc finger MYND-type containing 8
**38**	APC	APC, WNT signaling pathway regulator
**39**	ATXN7L3	ataxin 7-like 3
**40**	UBA2	ubiquitin-like modifier activating enzyme 2
**41**	XYLB	Xylulokinase
**42**	HES1	hes family bHLH transcription factor 1
**43**	CCDC13	coiled-coil domain containing 13
**44**	FOXA1	forkhead box A1
**45**	LRRC3B	leucine-rich repeat containing 3B
**46**	NEK8	NIMA related kinase 8
**47**	QARS1	glutaminyl-tRNA synthetase 1

a Adj rank = adjusted rank.

### Identified *PPARG* regulators are linked to MIBC

**Figure 3:**
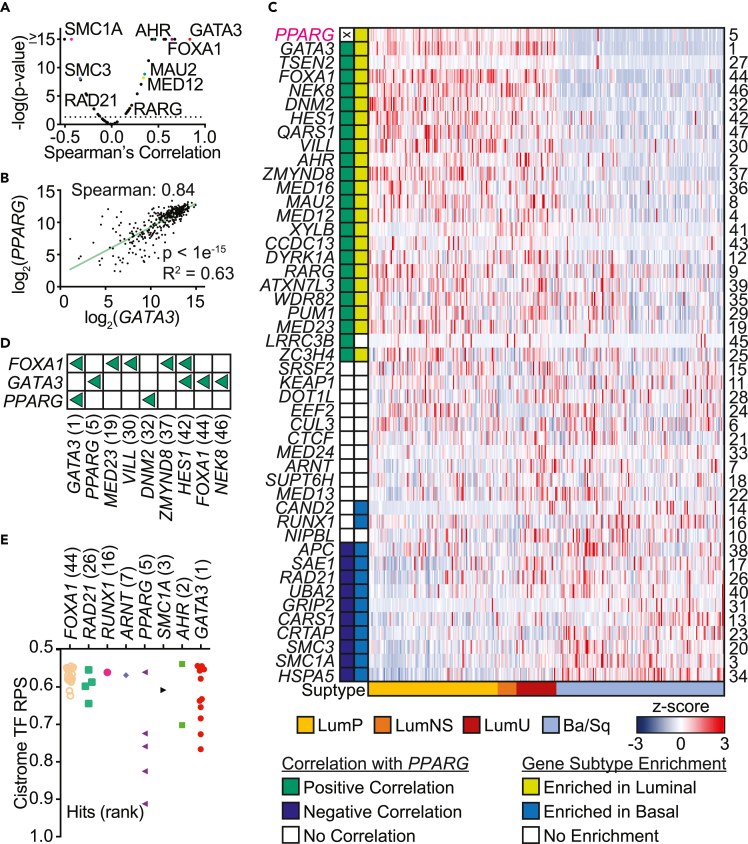
Top positive regulator hits are enriched in luminal MIBC (A–C) The top hits from the GFP^lo^ samples (putative positive regulators) were correlated with *PPARG* gene expression in luminal and basal MIBC tumor samples, excluding samples with *PPARG* copy number amplification (TCGA, n = 303). (A) Volcano plot represents Spearman’s coefficients relative to adjusted two-sided p-value for each gene. Highlighted (colored and named) are some of the hits relevant to identified functional clusters. The dotted line indicates p = 0.05. (B) Correlation of *GATA3* and *PPARG* mRNA levels is shown. (C) Heatmap displaying mRNA expression (RNAseq V2 RSEM), as represented by *Z* score, for each of the top 47 hits. *PPARG* is highlighted in pink. Rows are ordered from top to bottom by decreasing Spearman’s correlation coefficient based on correlation of expression of each gene with *PPARG*. Adjusted gene rank is indicated on the right, gene IDs are indicated on the left. Subtyping of each sample was performed using the MIBC consensus classifier, and are indicated on the bottom of the plot (LumP = luminal papillary, LumNS = luminal nonspecified, LumU = luminal unstable, Ba/Sq = basal/squamous). Positive (green) and negative (dark blue) correlation of the indicated genes with *PPARG* (adj. p < 0.05) are indicated along the left. White boxes indicate no significant correlation. Positive gene enrichment in luminal (yellow-green; LumP, LumNS, LumU) or basal (blue; Ba/Sq) tumors is indicated along the left. (D) Presence of the indicated genes (left) within the regulons^18^ of PPARγ, GATA3, or FOXA1 (top) is indicated with a green triangle (positive regulation). (E) Regulatory potential score (RPS) as calculated by the BETA algorithm from the Cistrome database. Each point within the rows represents a unique cancer or tissue type. (D and E) Adjusted hit rank is indicated in brackets.

In order to investigate potential regulators of *PPARG* expression with relevance in MIBC, we correlated the expression of the top hits with *PPARG* in a cohort of luminal (LumP, LumNS, and LumU) and basal (Ba/Sq) tumors from the TCGA database that lacked *PPARG* copy number amplification (n = 303). Of the 46 top positive hits (excluding *PPARG*), 33 significantly correlated (adj. p < 0.05) with *PPARG* expression, and 23 of them were in a positive direction (Figures 3A and 3C). Of note, the top-ranking gene of our screen, *GATA3*, also exhibited the highest Spearman correlation value in the TCGA cohort (Figures 3A and 3B). Consistent with high *PPARG* expression being a feature of luminal MIBC, the majority of the top positive hits that positively correlated with *PPARG* (22/23) were enriched in luminal (LumP, LumNS, and LumU) compared with basal (Ba/Sq) MIBC tumors (Figure 3C). A similar analysis looking at negative regulators of *PPARG* expression revealed 33 hits that significantly (adj. p < 0.05) correlated with *PPARG* (Figure S5A). Of the hits, 24 were negatively correlated with *PPARG*, and 25 were enriched in basal tumors compared with luminal in MIBC (Figure S5B).

We next examined the putative target gene sets (regulons)^18^ of three main drivers of the luminal biology in MIBC (*PPARG*, *GATA3*, and *FOXA1*) and found nine of the hits among the target genes (Figure 3D). Interestingly, PPARγ and GATA3 are present in each other’s regulon, suggesting the possibility of a positive feedback loop for the two transcription factors. Furthermore, many of the hits identified as positive regulators of *PPARG* gene expression by our screen fall within the regulons of GATA3 and FOXA1, suggesting a potential indirect role for these transcription factors, in particular FOXA1, whose putative regulon does not include *PPARG* (Figure 3D).

The Cistrome database provides information regarding the potential of a protein to affect the expression of a specific gene of interest.^38^ This probability is given through a regulatory potential score (RPS) in which chromatin immunoprecipitation sequencing (ChIP-seq) data of transcription factors or chromatin regulators are integrated with differential gene expression analyses through the BETA algorithm to infer direct target genes.^39^ Of the positive hits, eight appeared among the potential regulatory factors of *PPARG* expression with a positive RPS score (Figure 3E). Here again, GATA3 was among the most promising candidates.

### Distinct cellular processes contribute to the regulation of *PPARG* gene expression

**Figure 4:**
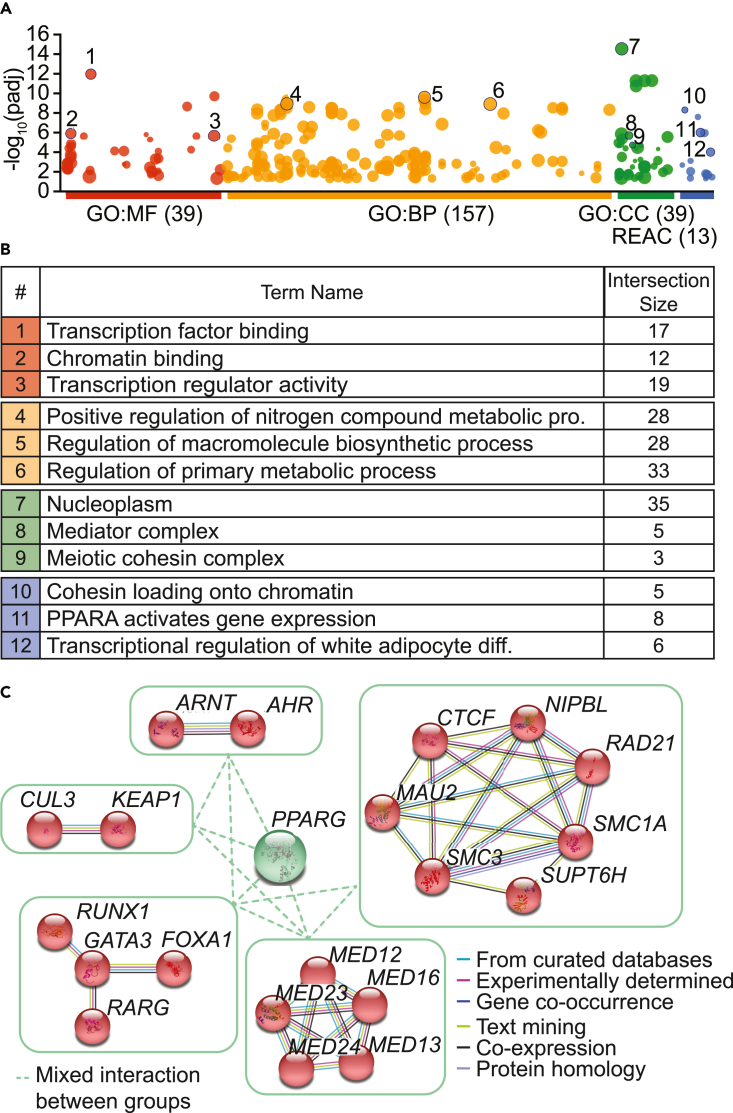
Putative top positive regulator hits are composed of distinct functional groups (A and B) Functional enrichment analysis of the top 47 hits from the GFP^lo^ group was performed with the g:GOSt tool. (A) Functional terms (colored circles) are grouped on the x axis and color-coded by data sources (GO = Gene Ontology, REAC = Reactome, BP = biological process, MF = molecular function, CC = cellular component). Bracketed numbers indicate how many significantly enriched terms are from this source. Adjusted enrichment p-values are plotted on the y axis. The circle sizes are proportional to the corresponding term size. Location of terms is fixed on the x axis and terms from the same data source are closer to each other. Numbered circles indicate some of the relevant terms, and (B) detailed information (term name and number of top hits intersecting with the term) are described in the table. (C) Diagram shows interactions between the top hits as determined by the STRING database. Highlighted are the main relationships between some of the top hits, which are clustered into groups that are part of a protein complex or are involved in similar functions.

We next sought to gain insight into the biological mechanisms that regulate *PPARG* gene expression as identified by the screen. To do this, enrichment analysis was performed using the g:GOSt tool provided by the g:Profiler platform that utilizes Gene Ontology (GO) terms and the biological pathway database, Reactome (REAC), to identify the biological pathways enriched in the list of putative positive regulators (Figures 4A and 4B). This analysis showed that most of the top positive hits were localized within the nucleus and had transcriptional regulatory activity. Moreover, many were involved in processes known to be regulated by PPARγ, such as macromolecule biosynthesis and cellular differentiation programs.^15^ Similar analysis of the top 50 negative regulator hits suggested that they were mainly localized in the nucleus, and were involved in activities related to cell cycle regulation (Figures S6A and S6B).

Finally, we aimed to identify functional groups that could reveal potential interdependencies between our hits. To do this, we utilized STRING, a biological database of known and predicted protein-protein interactions, and identified five major functional groups within the top positive regulator hits (Figure 4C). The groups included a cluster of transcription factors important in the biology of luminal bladder cancer (GATA3 and FOXA1), elements of the cohesin and mediator complexes involved in regulation of transcription and chromatin structure maintenance, the aryl hydrocarbon receptor involved in the detoxification from xenobiotic chemicals, and the KEAP1/CUL3 E3-ubiquitin ligase complex, a key player in the oxidative stress response. Similar analysis of the top negative hits identified a major cluster of 18 proteins involved in cell replication, and DNA synthesis and maintenance systems. Other smaller subgroups involved in RNA processing, protein folding, and general transcription factor scaffolding components were also identified (Figure S6B). Together, these data suggest a possible mechanism of *PPARG* repression related to cell cycle progression.

### *PPARG* gene expression is regulated by GATA3 in luminal bladder cancer

**Figure 5:**
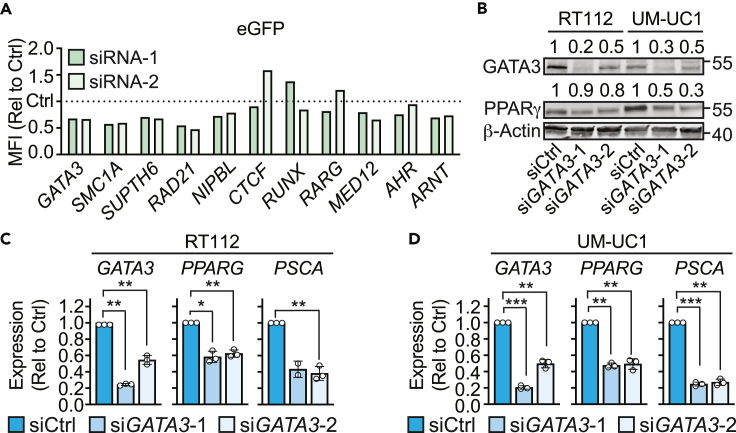
GATA3 is a positive regulator of *PPARG* gene expression in bladder cancer (A) RT112-PPARγ^GFP^ cells were treated with siRNAs targeting the indicated genes or a non-targeting siRNA (Ctrl). Median fluorescence intensity of eGFP was assessed by flow cytometry. Data are expressed as fold change compared to control scramble siRNA (Ctrl, dotted line). Data are representative of 2–3 independent repeats. (B–D) GATA3 was knocked down in RT112 and UM-UC1 cells by siRNA. (B) GATA3 and PPARγ protein levels were assessed by Western blot and β-actin was used as a loading control. Numbers above represent relative intensity of the band compared with the corresponding siCtrl sample. Each sample was first normalized relative to its corresponding β-actin band. Numbers on the right indicate molecular weight (kDa) of closest molecular weight marker. (C and D) mRNA levels of the indicated genes in (C) RT112 and (D) UM-UC1 cells were assessed by RT-qPCR. Data plotted as mean of 3 independent repeats with standard error of mean. Graphs represent mRNA expression relative to siCtrl. Each experimental sample (siGATA3) was normalized to the corresponding siCtrl sample in each experiment. Statistical significance was calculated using one-way ANOVA. ∗p < 0.05, ∗∗p < 0.01, ∗∗∗p < 0.001.

We next sought to validate a subset of the hits identified in the knockout screen. The targets were chosen among the top-ranking hits focusing on those at the core of the most relevant identified functional clusters. As a first proof-of-principle, the selected candidate genes were knocked down in RT112-PPARγ^GFP^ cells by siRNA and changes in eGFP expression were assessed by flow cytometry. Knocking down *GATA3*, *SMC1A*, *RAD21*, *SUPT6H*, *MED12*, and *ARNT* led to a consistent decreased in eGFP expression compared to control siRNA (Figure 5A).

Of these hits, GATA3 is also associated with luminal MIBC^23^ and had the strongest correlation with *PPARG* expression in patient samples (Figures 3A–3C). To validate the effect of GATA3 perturbation on *PPARG* gene expression, *GATA3* was knocked down in RT112 and UM-UC1 cells, which led to a 38%–52% decrease of *PPARG* mRNA, and a 10%–70% decrease in PPARγ protein by Western blot (Figures 5B–5D). Moreover, this also resulted in a similar decrease in the expression of the PPARγ target gene *PSCA* in both cell lines (Figures 5C and 5D). These data indicate that *PPARG* gene expression is affected by GATA3 in luminal bladder cancer.

### The cohesin complex contributes to the regulation of *PPARG* gene expression in luminal bladder cancer

**Figure 6:**
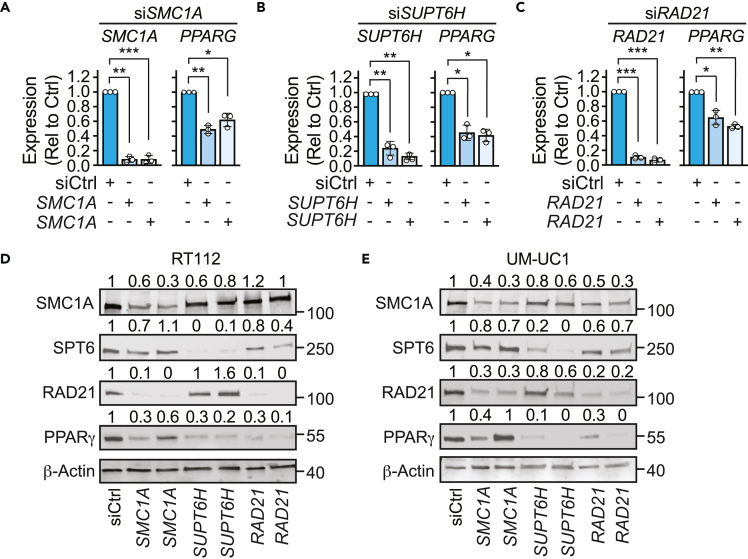
The cohesin complex promotes *PPARG* gene expression in bladder cancer *SMC1A*, *SUPT6H*, or *RAD21* were knocked down in RT112 and UC1 cells by siRNA. (A–C) mRNA expression levels of the indicated genes in RT112 cells were assessed by RT-qPCR. Data plotted as mean of 3 independent repeats with standard error of mean. Graphs represent mRNA expression relative to siCtrl. Each experimental sample was normalized to the corresponding siCtrl sample in each experiment. Statistical significance was calculated using one-way ANOVA. ∗p < 0.05, ∗∗p < 0.01, ∗∗∗p < 0.001. (D and E) Expression of the indicated proteins in (D) RT112 and (E) UM-UC1 cells were assessed by Western blot, with β-actin used as a loading control. Numbers above represent relative intensity of the band compared with the corresponding siCtrl sample. Each sample was first normalized relative to its corresponding β-actin band. Numbers on the right indicate molecular weight (kDa) of closest molecular weight marker. SPT6 is encoded by *SUPT6H*. Images are representative of two (UM-UC1) or three (RT112) independent repeats.

Several of the putative positive regulators of *PPARG* expression are known to participate in chromatin remodeling and maintenance functions. Many of these (*e*.*g*. SMC1A and RAD21) are direct components of the cohesin complex while others, such as SPT6 (encoded by *SUPT6H*), are functionally related.^40^^,^^41^ *SMC1A*, *RAD21*, and *SUPT6H* were each silenced by siRNA in RT112 cells, and *PPARG* mRNA was quantified by RT-qPCR. KD of *SMC1A*, *RAD21*, and *SUPT6H* each resulted in a significant (35%–58%) decrease in *PPARG* gene expression (Figures 6A–6C). Consistent with this, siRNA targeting *RAD21* and *SUPT6H* in RT112 and UM-UC1 cells led to a reduction in PPARγ protein compared to the scrambled siRNA control (Figures 6D and 6E). Despite a similar reduction of *PPARG* mRNA expression following KD of each of the three genes, only loss of *RAD21* and *SUPT6H*, but not *SMC1A*, led to a consistent robust reduction in PPARγ protein. In addition, KD of *SMC1A* also led to a decrease in RAD21 protein, suggesting a feedback loop between these two proteins, and that SMC1A is required for RAD21-dependent reduction in PPARγ (Figures 6D and 6E). In summary, these data suggest that the cohesin complex promotes expression of *PPARG* in luminal bladder.

## Discussion

Finding a clear and reliable phenotype is the first major step in large-scale gene expression screens. Historically, indirect reporter systems have been used, in which reporter genes, such as luciferase, eGFP, β-galactosidase, or alkaline phosphatase, are linked to a putative promoter for the gene in study.^42^^,^^43^ Despite the fact that this approach is still widely used, it carries intrinsic drawbacks that decrease the biological relevance of its findings. By relying on the random insertion of the reporter cassette in the genome, common reporter systems can result in the unwanted disruption of genes potentially relevant for the phenotype in analysis. Another risk is the possible insertion of the reporter in silent or highly transcribed genomic regions that can confound the readout. Moreover, this technique does not consider the full complexity of gene regulation, which is fine-tuned by a combination of promoter sequences, adjacent DNA regions, enhancers, epigenetics, and other highly context-dependent regulatory mechanisms.^44^^,^^45^^,^^46^^,^^47^ Finally, a specific promoter sequence needs to be verified *a priori*, and this is frequently assessed in experiments carried out in different biological systems. Thus, the usage of an exogenous and simplistic promoter-reporter system can lead to generation of misleading and non-biologically relevant data.

CRISPR knockin technology is a powerful tool to deliver an exogenous reporter in a specific site within the genome, allowing for expression of the reporter under the same regulation as the gene of interest without the requirement for previous promoter knowledge or other assumptions. This can then be combined with CRISPR knockout technology to perform a highly specific and large-scale genome-wide screen^48^^,^^49^^,^^50^. Given the complexity of functions and regulatory mechanisms of PPARγ, which are highly variable within distinct biological contexts, it was imperative to use a reporter system knocked into the endogenous locus of a cell type relevant to our disease of interest. We therefore utilized CRISPR knockin technology to create a transgenic luminal bladder cancer cell line engineered to express *GFP* and *PPARG* proportionally, so that fluorescence could be used as a readout to identify endogenous regulators of *PPARG* expression. With this reporter line, we performed a high-throughput genome-wide CRISPR knockout screen using the Brunello lentiviral library which comprises 76,441 unique sgRNAs targeting 19,114 coding genes and an additional 1000 non-targeting sgRNAs for control. Compared to previously published sgRNA collections, the Brunello library was designed with optimized rules to improve on-target activity and lower off-target effects.^51^ Together, these systems provided a robust method of identifying biologically and clinically relevant regulators of *PPARG* gene expression.

In the GFP^lo^ group, we found potential positive regulators of *PPARG* expression which correlated with *PPARG* mRNA level and were enriched in the luminal subtype. This, combined with the identification of functional clusters within our list of hits, highlighted the efficacy of the screen. Correlation of our hits with *PPARG* in MIBC samples was used as a first means to filter our screen results to include those with likely relevance to bladder cancer *in vivo*. However, this may have falsely excluded hits with biological significance as a result of potential adverse effects for the overall tumor survival *in vivo*. Furthermore, the presence of mixed populations of cells in the whole-tissue RNAseq data could confound the analysis since different cell types express different amounts of each of the genes (including *PPARG*). Consistent with this, although expression of *SMC1A* and *RAD21* both negatively correlated with *PPARG* in MIBC samples, they were validated as positive regulators of *PPARG* gene expression in two independent cell lines. Therefore, further analysis and validation of the hits identified in our screen has the potential to generate significantly more biological insight into regulators of *PPARG* expression specifically in bladder cancer cells.

We choose to focus our validation on positive regulators of *PPARG* as they had the most obvious potential relevance for luminal MIBC. By using a luminal bladder cancer cell line with high *PPARG* expression, we likely selected a system that lacked activity of many of the regulatory mechanisms that actively suppress *PPARG* gene expression, therefore limiting our ability to identify these mechanisms using a gene knockout system. Furthermore, pathway enrichment analysis suggested the involvement of the top hits in broad cellular processes including cell cycle progression, RNA splicing, and protein translation. Although these mechanisms could potentially alter PPARγ expression, they could also be an artifact of the screen by reducing cell duplication and protein turnover, leading to an accumulation of eGFP within the cells. Nonetheless, within our top negative hits are some that have been previously identified as important in basal bladder cancer, including CDK6 and p63 (encoded by *TP63*).^52^ p63 is a known driver of the basal subtype and it inhibits genes associated with epithelial differentiation in normal human urothelial cells, including *PPARG*.^17^^,^^53^^,^^54^ Although these findings warrant further investigation, an analogous screen performed on a basal cell line expressing low PPARγ level could support identification of relevant inhibitors of PPARγ expression. Alternatively, a CRISPR-based gene activator screen could be performed using the RT112-eGFP reporter cell line. Future studies that combine whole-genome activator and knockout screens in both luminal and basal bladder cancer will provide a comprehensive framework of the various positive and negative regulatory mechanisms that are necessary and/or sufficient to modulate *PPARG* gene expression in MIBC.

GATA3 is a member of the GATA-binding protein family of transcription factors that recognize the DNA consensus sequence A/T-GATA-A/G.^55^ GATA3 has been extensively studied for its role in hematopoietic tissues and, in particular, for its central role in the development in multiple immune cell lineages.^56^^,^^57^ It has also been implicated in the proper development of numerous tissues including skin, kidney, rectum, breast, and bladder.^58^ In the ductal epithelium of the mammary gland, GATA3 is highly expressed in the luminal cells while it is absent from the basal layer containing a pool of uncommitted progenitor cells.^59^^,^^60^ An analogous expression pattern appears in normal bladder, and in MIBC.^33^ GATA3 is well characterized to be associated with PPARγ and FOXA1 as an urothelial differentiation marker and driver of the luminal MIBC subtype. Counterintuitively, few studies have investigated GATA3 as a regulator of PPARγ expression in the context of urothelial carcinoma. In our genome-wide knockout screen, GATA3 ranked first as a possible *PPARG* regulator, and siRNA knockdown of GATA3 resulted in decreased PPARγ at the mRNA and protein level. Investigations into the regulation of *PPARG* by GATA3 have reported contrasting observations. A report on differentiation of pre-adipocytes showed a role of GATA3 in suppressing *PPARG*, while overexpression of the same transcription factor in buccal epithelial cells failed to display any effect.^61^^,^^62^ Similarly, *PPARG* did not emerge as significantly upregulated gene by GATA3 overexpression in 5637 basal bladder cancer cells.^33^ However, 5637 cells contain a CASC15-PPARγ fusion,^18^ which may alter the normal regulation of *PPARG* expression. Furthermore, these discrepancies underline the importance of the biological context for GATA3 target selection. Given the variety of tissues and cell types in which GATA3 plays a crucial role, it is not surprising that context-dependent differential expression of binding partners alters its activity. In support of this hypothesis, a study conducted on normal human urothelial cells that silenced *GATA3* gene expression reported a reduction in *PPARG* expression, in agreement with our findings.^54^

We identified and validated GATA3 as a positive regulator of *PPARG* in bladder cancer. Our data are supported by a recent study that performed GATA3 ChIP-Seq on the luminal bladder cancer cell line RT4 and identified GATA3 binding to enhancer regions of *PPARG*.^63^ Consistent with GATA3 regulating *PPARG* by binding DNA at distal sites, identification of components of the mediator and cohesin complexes as hits in our screen suggests that chromatin looping and binding of transcription factors at distal enhancer sites are involved in the regulation of *PPARG*.^64^ Alternatively, GATA3 may indirectly regulate PPARγ by altering cell differentiation or luminal phenotypes. In agreement with this, FOXA1, a luminal differentiation driver together with PPARγ and GATA3, was also identified as a top hit. Further experiments will elucidate the exact mechanism by which GATA3 drives *PPARG* expression.

Chromatin remodelers and components of the cohesin complex have been less well studied in the context of bladder cancer. Cohesin components including SMC1A, SMC3, RAD21, NIPBL, MAU2, and other functionally related proteins, such as MED and CTCF factors, ranked highly in our screen, suggesting a deep involvement of chromatin structuring elements in the regulation of *PPARG* gene expression. Here, we reported a strong inhibition of *PPARG* expression following loss of SMC1A or RAD21. Not all members of the cohesin complex were identified as regulators of *PPARG* expression, which suggests that distinct cohesin components, or their relative abundance, may confer transcriptional target specificity to the whole complex. Moreover, the observed downregulation of RAD21 protein upon *SMC1A* silencing might have unveiled an intrinsic regulatory mechanism of the cohesin members, which indirectly affects PPARγ expression. In addition, we reported that the histone chaperone SPT6 is important in sustaining *PPARG* expression bladder cancer cells. This agrees with data from human mesenchymal stem cells in which downregulation of *SUPT6H* results in reduction of *PPARG* expression, which was essential in their differentiation.^65^ Similarly, the cohesin complex is involved in cell differentiation, particularly during hematopoiesis^66^ and thymocyte development.^67^ Altogether, we identified regulators of *PPARG* gene expression which, despite their diverse molecular mechanisms, all have important roles in cellular differentiation as a common feature. Our data place PPARγ downstream of these major differentiating factors, and further experimentation will shape the full details of the pathways involved.

Overall, we have generated a reporter system to monitor changes in *PPARG* gene expression in bladder cancer cells. Using this system, we performed a genome-wide CRISPR knockout screen that generated a robust dataset valuable to the study of PPARγ and bladder cancer biology. We also validated four hits, spanning distinct biological processes, that support the validity and biological relevance of the reporter system and whole-genome knockout screen data presented here. Finally, these data support the utility of the reporter system to assess potential therapies targeting pathways that regulate *PPARG* gene expression in luminal bladder cancer.

### Limitations of the study

A high level of *PPARG* mRNA is a common feature of luminal MIBC.^16^ However, the upstream components that regulate *PPARG* expression in these tumors remain unclear. We therefore sought to identify putative regulators of *PPARG* mRNA expression by performing a genome-wide CRISPR knockout screen in a luminal bladder cancer reporter cell line. The major aim of the study was to identify potential regulators of *PPARG* gene expression in bladder cancer and to generate a publicly available resource to drive and support future studies. Additional validation was used to provide evidence of the potential biological relevance of the data, but the *in vivo* relevance of the hits was not dissected in detail.

Although we confirmed some key hits in a second cell lines, the use of a single cell line to perform the screen constitutes a limitation of the study. Furthermore, the use of a luminal cell line with high *PPARG* expression likely limited our ability to identify negative regulators of *PPARG* expression, as many of them would not be active in our system. These limitations could be overcome in future studies by increasing the number of cell lines used for the screen including basal cell lines with low *PPARG* expression. Alternatively, a gene activator screen could be used in the luminal cell lines to better identify negative regulators of *PPARG* gene expression.

An additional limitation of our study is the limited *in vivo* data to support our findings. Attempting to address this, we assessed the correlation of the mRNA levels of our screen hits with *PPARG* in the TCGA patient cohort. However, there are a number of caveats to this approach. One of these caveats is that bulk gene expression data from tissue samples include gene expression from a heterogeneous population of cells comprised not only tumor cells but also other stromal cells such as fibroblasts and immune cells. The mechanisms of regulation of *PPARG* gene expression may be different in different cell types, which would therefore confound the comparison between data from a cell line and whole tissue. Another caveat to this analysis is the use of gene expression correlation to validate hits from a gene knockout. The screen described here used gene knockouts to identify putative regulators of *PPARG* gene expression without information about the relative expression of each of the hits. Although useful as a means to support the overall validity of our findings, there is no requirement for correlation between the level of mRNA encoding for proteins that regulate expression of a gene, and the mRNA level of that target gene. Therefore, future studies that carefully validate the putative regulators *in vivo*, such as through orthotopic cell line or patient-derived xenograft models, are required.

Overall, despite the limitations of the study, the data presented here fulfill the major aim of our study, and provide a valuable resource for the community that can serve to support future work investigating PPARγ biology in bladder cancer.

## STAR★Methods

### Key resources table

**Table:** 

REAGENT or RESOURCE	SOURCE	IDENTIFIER
**Antibodies**		

Monoclonal Anti-β-Actin antibody produced in mouse (clone AC-74)	Millipore Sigma	Cat# A2228; RRID:AB_476697
GATA-3 (D13C9) XP® Rabbit mAb	Cell Signaling Technologies	Cat# 5852; RRID:AB_10835690
PPARγ (C26H12) Rabbit mAb	Cell Signaling Technologies	Cat# 2435; RRID:AB_2166051
RAD21 Polyclonal Antibody	ThermoFisher Scientific	Cat# A300-080A; RRID:AB_2176615
SMC1A Polyclonal Antibody	ThermoFisher Scientific	Cat# A300-055A; RRID:AB_2192467
SUPT6H Polyclonal Antibody	ThermoFisher Scientific	Cat# A300-801A; RRID:AB_577215

**Bacterial and virus strains**		

NEB Stable competent *E*. *coli* (High efficiency)	New England Biolabs	Cat# C3040H
ElectroMAX Stbl4 Competent Cells	Thermo Scientific	Cat# 11635018

**Chemicals, peptides, and recombinant proteins**		

cOmplete ULTRA Tablets, Mini, EDTA-free, EASYpack Protease Inhibitor Cocktail	Millipore Sigma	Cat# 5892791001
PhosSTOP	Millipore Sigma	Cat# 4906837001

**Critical commercial assays**		

X-tremeGENE HP DNA transfection reagent	Millipore Sigma	Cat# 6366244001
Lipofectamine™ RNAiMAX Transfection Reagent	ThermoFisher Scientific	Cat# 13778075
CellTiter-Glo Luminescent Cell Viability Assay	Promega	Cat# G7570
Quant-iT dsDNA HS assay kit	ThermoFisher Scientific	Cat# Q33120
Q5 High-Fidelity 2X Master Mix	New England Biolabs	Cat# M0492L
Qiaquick PCR Purification Kit	Qiagen	Cat# 28104
AMPure XP Reagent	Beckman Coulter	Cat# A63881
Bioanalyzer High Sensitivity DNA Kit	Agilent	Cat# 5067-4626
FastAP Thermosensitive Alkaline Phosphatase	ThermoFisher Scientific	Cat# EF0651
T4 Polynucleotide Kinase	New England Biolabs	Cat# M0201S
LunaScript RT SuperMix Kit	New England Biolabs	Cat# E3010S
Luna Universal qPCR master mix	New England Biolabs	Cat# M3003X

**Deposited data**		

CRISPR screen sequencing data	This manuscript	NCBI SRA BioProject ID: PRJNA936268

**Experimental models: Cell lines**		

RT112	Lab of Dr. David McConkey	RRID:CVCL_1670
UM-UC1	Lab of Dr. David McConkey	RRID:CVCL_2743
HEK293-FT	ThermoFisher Scientific	Cat# R70007; RRID:CVCL_6911

**Oligonucleotides**		

Primers for RT-qPCR, see Table S2	This manuscript	N/A
NGS Primers, see Table S3	Joung et al.^68^	N/A
Reporter Validation PCR Primer (eGFP): CTTGTACAGCTCGTCCATG	This manuscript	N/A
Reporter Validation PCR Primer (HR-L): GTAAAATTGTCCTGGAACCCTGTG	This manuscript	N/A
Reporter Validation PCR Primer (HR-R): AGAGCGTGGCGGAACTTATG	This manuscript	N/A
Reporter Validation PCR Primer (Neo): CATCGACTGTGGCCGGCT	This manuscript	N/A
CRISPR targeting oligonucleotide (PPARG-A-Fwd): CACCGTGGCATCTCTGTGTCAACCA	This manuscript	N/A
CRISPR targeting oligonucleotide (PPARG-A-Rev): AAACTGGTTGACACAGAGATGCCAC	This manuscript	N/A
CRISPR targeting oligonucleotide (PPARG-B-Fwd): CACCGTTTCCTTTCAGAAATGACCA	This manuscript	N/A
CRISPR targeting oligonucleotide (PPARG-B-Rev): AAACTGGTCATTTCTGAAAGGAAAC	This manuscript	N/A
CRISPR targeting oligonucleotide (PPARGKO-1-Fwd): CACCGAATGCTGGAGAAATCAACTG	This manuscript	N/A
CRISPR targeting oligonucleotide (PPARGKO-1-Rev): AAACCAGTTGATTTCTCCAGCAATC	This manuscript	N/A
CRISPR targeting oligonucleotide (PPARGKO-2-Fwd): CACCGAGAACCTTCTAACTCCCTCA	This manuscript	N/A
CRISPR targeting oligonucleotide (PPARGKO-2-Rev): AAACTGAGGGAGTTAGAAGGTTCTC	This manuscript	N/A

**Recombinant DNA**		

Donor plasmid pPPARG^HR^-eGFP (pDonor-DTA-{hPPARG_LA}:{EGFP:T2A:Neo:P2A}:{hPPARG_RA})	VectorBuilder	Vector ID: VB181120-1027ysz
lentiCRIPSRv2	Addgene	Cat# 52961
CRISPR targeting plasmid (LentiCRISPRv2-PPARG A/B)	This manuscript	N/A
psPAX2 - Lentiviral packaging plasmid	Addgene	Cat# 12260
pMD2.G - VSV-G envelope expressing plasmid	Addgene	Cat# 12259
Human CRISPR Knockout Pooled Library (Brunello)	Addgene	Cat# 73179
PhiX Control v3	Illumina	Cat# FC-110-3001
siRNA, see Table S4.	This manuscript	N/A

**Software and algorithms**		

seqtk trimfq (version 1.2-r94)	NA	https://github.com/lh3/seqtk
Fastqc	Shen et al.^69^	https://www.bioinformatics.babraham.ac.uk/projects/fastqc/
BWA MEM	Robinson et al.^70^	https://github.com/lh3/bwa
MAGeCK version 0.5.7	Li et al.^37^	https://anaconda.org/bioconda/mageck
consensusMIBC R package	Kamoun et al.^23^	https://github.com/cit-bioinfo/consensusMIBC
NetworkAnalyst	Zhou et al.^71^	https://dev.expressanalyst.ca/ExpressAnalyst/uploads/TableUploadView.xhtml
Limma	Ritchie et al.^72^	https://doi.org/10.18129/B9.bioc.limma
Cor.test R function	NA	https://www.rdocumentation.org/packages/stats/versions/3.6.2/topics/cor.test
Cistrome database and toolkit for Cistrome data browser	Liu et al.^38^	http://dbtoolkit.cistrome.org/
g:GOST tool (g:Profiler platform)	Raudvere at al.^73^	https://biit.cs.ut.ee/gprofiler/gost
STRING	Szklarczyk et al.^74^	https://string-db.org/
FACSDiva v7	BD Biosciences	https://www.bdbiosciences.com/en-ca/products/software/instrument-software/bd-facsdiva-software
ImageJ	Schneider et al.^75^	https://imagej.nih.gov/ij/
FlowJo v10	BD Biosciences	https://www.flowjo.com/solutions/flowjo

### Resource availability

#### Lead contact

Further information and requests for resources and reagents should be directed to and will be fulfilled by the lead contact, Mads Daugaard (mads.daugaard@ubc.ca).

#### Materials availability

All plasmids and cell lines generated in this manuscript will be made available upon request. A material transfer agreement will be required prior to sharing of materials.

### Experimental model and subject details

#### Cell lines

Human bladder cancer cell lines were kindly provided by Dr. David J. McConkey (University of Texas, MD Anderson Cancer Center, Huston, Texas, USA). UM-UC1 (male, RRID:CVCL_2743) and RT112 (female, RRID:CVCL_1670) cells were authenticated in 2013 and 2019, respectively. HEK293-FT cells (female, RRID:CVCL_6911) were purchased from Thermo Fisher Scientific. UM-UC1 and RT112 cell were maintained in HyClone™ Minimum Essential Medium with Earle’s Balanced Salts (MEM/EBSS) + 10% FBS. HEK293-FT cells were grown in Dulbecco’s Modified Eagle’s Medium with high glucose (DMEM) + 10% FBS. Medium was supplemented with 500 μg/mL Geneticin (Gibco) to maintain expression of the viral T-antigen in HEK293-FT. To passage cells or collect them for experiments, cells were detached using Trypsin-EDTA solution. All cells were grown at 37°C with 5% CO_2_.

### Method details

#### Western blot

Total protein lysates were collected in RIPA buffer supplemented with protease (cOmplete™ ULTRA Tablets, Mini, EDTA-free, EASYpack Protease Inhibitor Cocktail, Sigma) and phosphatase (PhosSTOP™, EASYpack, Sigma) inhibitors. Lysate were sonicated (5 cycles, 10 seconds on, 15 seconds off) using a Bioruptor® 300 (Diagenode). Protein concentrations were quantified using the Pierce™ BCA Protein Assay Kit (ThermoFisher Scientific) and 40-100μg of protein was loaded for blot analysis after heat denaturation in 1X sample buffer (60mM Tris-HCl, pH 6.8, glycerol, 2% (w/m) SDS, 0.005% β-ME). Fractionation was carried out by SDS-PAGE and transferred to a polyvinylidene difluoride (PVDF) or nitrocellulose membrane (0.2 μm) by overnight wet transfer. Membranes were blocked in TBST (10 mM Tris, pH 8.0, 150 mM NaCl, 0.5% Tween 20) plus 5% nonfat milk for 1 hour at room temperature. Primary and secondary antibody incubations were performed overnight at 4°C and 1 hour at room temperature, respectively, at the recommended dilutions. Blots were developed according to the manufacturer’s recommendations with Clarity Max™ Western ECL Substrate (Biorad) for HRP-conjugated secondary detection, or with the Odyssey (Li-Cor Biosciences) system. ImageJ^75^ was used to quantify band intensities.

#### Plasmids

All plasmids were amplified in NEB Stable® competent *E*. *coli* (NEB) using the heat-shock transformation method. Briefly, 1ng of the plasmid was added into a vial of competent *E*. *coli*, incubated on ice for 30 minutes, heat-shocked for 30 seconds at 42°C, and then recovered on ice for 2 minutes. Next, 250 μl of SOC medium (Sigma) was added to each vial which was then incubated at 37°C for 1 hour in a shaking incubator. About 100 μl of the mixture was spread on a pre-warmed agarose plate containing 100 μg/ml ampicillin for transformant selection, and plates were incubated overnight at 37°C. Selected colonies were grown overnight in 5ml of Miller’s LB broth (Invitrogen) supplemented with ampicillin at 100 μg/ml. The plasmids were then harvested using the Monarch® Plasmid Miniprep kit (NEB) according to the manufacturer’s protocol.

pPPARG^HR^-eGFP donor plasmid was custom synthesized by VectorBuilder (Vector ID: VB181120-1027ysz). The plasmid was linearized using NotI restriction enzyme (NEB) according to the manufacturer’s instructions.

CRISPR plasmids targeting the 3′ *PPARG* region (LentiCRISPRv2-PPARG A/B) used for the creation of the reporter line were based on the lentiCRIPSRv2 (Addgene #52961). The target sequences were cloned into the above-mentioned plasmids using Dr. Feng Zhang laboratory’s protocol.^76^,^77^ Briefly, plasmids were digested with Esp3I (Thermo Scientific) restriction enzyme and extremities dephosphorylated with FastAP thermosensitive alkaline phosphatase (Thermo Fisher Scientific). Oligos encoding the target sequences were annealed and phosphorylated with T4 polynucleotide kinase (NEB). Digested plasmid and annealed oligos were ligated *in vitro* using Quick Ligase (NEB) and the resulting product used for bacteria transformation and amplification. For viral production (see below), psPAX2 (Addgene #12260) and pMD2.G (Addgene #12259) 2^nd^ generation lentiviral packaging plasmids were used.

Pre-synthesized human CRISPR knockout pooled library (Brunello) was originally purchased from Addgene (catalog #:73179) and amplified according to the published protocol.^68^ Briefly, 400ng of plasmid DNA (pDNA) were used for electroporation of 100 μL STBL4 electrocompetent cells (Thermo Scientific). Electroporation was performed using the Gene Pulser Xcell Microbial System (Bio-Rad), in 25 μl aliquots at 1.8kV, and the mix was then immediately transferred to 1mL of pre-warmed SOC medium. The 4 reactions were combined, SOC medium added to a final total volume of 10mL and incubated in a shaker for 1 hour at 30°C. Next, 2.5 mL of cells were plated onto each of four 500 cm^2^ bioassay plates (LB agar + 100 μg/mL Ampicillin) and incubated at 30°C for 18 hours. Finally, cells were harvested with 40 mL of cold LB, pelleted, and purified using the HiSpeed Plasmid Maxi Kit (Qiagen). Purified plasmid library was pooled and analyzed via Illumina MiSeq to ensure high quality and determine library distribution.

#### Cell transfection for transient plasmid expression

Transient transfection of the described plasmids was obtained using X-tremeGENE™ HP DNA transfection reagent (Sigma), according to the manufacturer’s indications. A 1:1 ratio was used for double transfection of lentiCRISPRv2-PPARG A/B and pPPARG^HR^-eGFP. A total of 20 μg of DNA was used to transfect a 15 cm plate of RT112 cells.

Lipofectamine RNAiMAX transfection reagent was used for the delivery of siRNAs. Reverse transfection was performed according to the manufacturer’s protocol using a final siRNA concentration of 10-50nM. Readout for siRNA experiments was performed 72–96 hours post transfection.

#### Lentiviral packaging

For general lentivirus production, HEK293FT cells were seeded at 4 × 10^6^ cell per 10cm plate, 24 hours before transient transfection with psPAX2 (4.5 μg), pMD2.G (1.5 μg) and the desired lentiviral plasmid (6 μg). 48 hours post transfection, lentivirus-containing supernatant was spun and filtered through a 0.45 μm filter to remove cellular debris.

For packaging of the pooled CRISPR library used in the whole genome knockout screen, the protocol published by Joung et al. was followed.^68^ Briefly, 1.8 × 10^7^ HEK293FT cells were plated in T-225 tissue culture flasks (Corning) 24 hours prior transfection. For each flask, packaging plasmids psPAX2 (23.4 μg), pMD2.G (15.3 μg) and pooled library (30.6 μg) were combined and transfected using Lipofectamine 3000 reagent as per manufacturer’s protocol. Medium was changed at 6 hours post transfection to avoid reagent toxicity and 2mM of caffeine 99% (Sigma) was added at 24 hours. At 48 hours post-transfection, virus-containing supernatant was then collected as above.

#### Transduction and cell culture and isolation for the CRISPR screen

Transduction was performed by centrifugation (a process known as spinfection^78^ and viral multiplicity of infection (MOI) was calculated as follows. 3 × 10^6^ cells per well were added into a 12 well plate with 1mL of MEM medium supplemented with 8 μg/ml polybrene. Different titrated amounts of viral supernatant were added to each well along with a no-transduction control. Spinfection was carried out by spinning the plate at 1,000 g for 2 hours at 37°C followed by overnight incubation at 37°C. Cells were then collected and each condition split into 4 wells of a 96-well clear-bottom black tissue culture plate (n = 6) (Corning). Half of the wells were treated with puromycin (for which resistance is carried by the packaged plasmid to be delivered) at a final concentration of 0.6 μg/ml. Cells were then cultured for 7 days replacing the medium with or without treatment every 2–3 days until cell viability was quantified in each condition using CellTiter Glo® (Promega) according to manufacturer’s protocol. MOI was finally calculated as a ratio between the average luminescence (viability) of antibiotic treated and untreated replicates. Virus volume yielding an MOI closest to 0.3 was used for large-scale screening.

To maintain a final library coverage of 1000X and aiming for 50% extra coverage to account for losses, for the large scale CRISPR screen we initially transduced 4.15 × 10^8^ RT112 cells. Spinfection were performed as described above, with cells plated at a density of 3 × 10^6^ cells per well in 12-well plates. 24 hours after spinfection, cells were trypsinized, pooled and then distributed into 500 cm^2^ tissue culture-treated plates (n = 24) at 1.73 × 10^7^ cells/plate. This seeding density was calculated by dividing 5.2 × 10^6^ cells/plate by the 0.3 MOI. 5.2 × 10^6^ cells/plate is the maximum number of RT112 cells that, plated on a 500 cm^2^ plate, will not grow over 90–95% confluency in a 7-day incubation period required for our screen. Puromycin selection (0.3 μg/ml) was added to each plate and maintained for 7 days, refreshing growth medium every 48 hours. After selection, cells were collected, resuspended in PBS + 2%FBS + 2.5mM EDTA + 200ng/ml propidium iodide. Cells were sorted by FACS with a BD FACSAria III or BD FACSAria Fusion (BD Biosciences) according PI exclusion (live cells) and eGFP fluorescence intensity. Sorted cells were pelleted and genomic DNA (gDNA) was then isolated using the Quick-DNA Midiprep Plus Kit (Zymo Research).

#### PCR amplification and NGS sequencing for CRISPR screen

Genomic DNA was extracted from cells sorted for the genome-wide CRISPR knockout screen and was processed for sequencing according to Joung et al.^68^ with some adjustments from the UBC Sequencing and Bioinformatics Consortium. Briefly, gDNA samples were first measured using a Quant-iT dsDNA HS assay kit on a Qubit fluorometer (Life Technologies). For each sample, individual 50 μl PCR reactions were set up using ∼3 μg DNA, 25 μl Q5 High-Fidelity 2X Master Mix (NEB) and 0.25 μM of each primer. Enough reactions were set up to process all of the extracted gDNA. To minimize amplification variation, reactions were set up as a single master mix for each sample. All samples used the same forward (fw) primers (an equimolar mix of 10 individual primers with staggered based) and a different reverse (rv) primer to enable barcoding. Amplification conditions were as follows: 3 min at 98°C; 22 cycles of 10 s at 98°C, 10 s at 63°C, and 25 s at 72°C; and a final elongation of 2 min at 72°C. For each sample, PCR reactions were pooled and purified using Qiaquick PCR purification columns (Qiagen), according to manufacturer’s instructions followed by concentration using AMPure XP beads (Beckman Coulter) at a ratio of 1.8X beads:DNA. Final library quality control was performed on a Bioanalyzer High Sensitivity DNA Chip (Agilent) and quantified using Qubit fluorometry. Libraries were pooled and sequenced over two NextSeq High Output flow cells (Illumina), generating single-end 80 bp reads. Amplicon library sequencing was performed with a 20% PhiX spike-in to compensate for low base diversity.

#### Computational analysis

The computational analysis of the CRISPR screen included counting and statistical tests for the assessment of guide and gene level significance, as well as quality control for sequencing, library representation and the reproducibility of results from replicate experiments. sgRNA sequences were staggered within each read to increase library complexity from 43 bases 5` to the start of the 80-base read. Therefore, reads were trimmed using seqtk trimfq (version 1.2-r94) with a trim of 42 bases from the left (-b) and 8 bases from the right (-e). Fastqc^69^ was used to assess quality metrics with respect to the raw sequencing reads before trimming. A reference FASTA of the CRISPR library was constructed by prepending and appending plasmid backbone bases to each guide sequence, 70 and 42 bases, respectively. Contig names were assigned with annotations of the gene name, a unique identifying index number, and the targeted exon number of each guide from the library bed file provided. After masking bases with a quality score less than 10, the reads were aligned to the reference using BWA MEM^70^ with a minimum seed length (-k) of 8 and a minimum alignment score (-T) of 15. Reads with a map quality (MAPQ) less than 6 were discarded. Each contig represented in the alignment was then counted. The normalized read representation and distribution with respect to each gRNA, gene, sample, and experiment was then evaluated and compared. These metrics were also compared to previously published experiments and within consultations made with the library vendors, BC Cancer’s Genome Sciences Centre, and UBC’s Sequencing and Bioinformatics Consortium. After determination that replicate GFP^tot^ samples were equivalent, the experiments were merged to increase statistical power. The merged groups were median normalized and MAGeCK version 0.5.7^37^ was used to produce guide and gene rankings using the MAGeCK Robust Rank Aggregation (RRA).

From the MAGeCK RRA test results, an additional heuristic filter was applied to remove false positive results. In each experimental group, enriched sgRNAs were organized according to an adjusted rank, calculated as the difference between the derived negative and positive MAGeCK ranks. This modified rank removed the subset of genes which had statistically significant high scores in both the lists for positive and negative enrichment derived from MAGeCK. Screening scores ranked within the top 50 and with an FDR<0.01 were further examined. Top hits were subjected to database analyses and manual curation of the literature before further experimental validation.

#### Bioinformatics analysis

RNAseq data for a panel of 30 urothelial cancer cells were downloaded though Gene Expression Omnibus (GEO, GSE97768) and subtypes were assigned based on the analysis from Robertson et al.^18^

Annotated patient mRNA expression data for correlation analysis were downloaded through cBioPortal from the TCGA MIBC cohort (TCGA, Cell 2017).^18^ Samples with *PPARG* copy number amplification were identified based on analysis from Robertson et al.^18^ and were excluded from the analysis. Patient samples were classified into six molecular subtypes (luminal papillary (LumP), luminal non-specified (LumNS), luminal unstable (LumU), stroma-rich, basal squamous (Bs/Sq) and neuroendocrine-like (NE-like)) using the *consensusMIBC* R package developed by Kamoun et al.*.*,^23^ and NE-like and stroma-rich samples were excluded to focus on the purest urothelial samples. Differential gene expression analysis was performed to assess enrichment of the positive and negative regulator hits (97 genes) in luminal (LumP, LumNS, and LumU) versus basal (Ba/Sq) samples using the NetworkAnalyst web-based tool^71^. The low abundance filter was set to 4 and the variance filter set to 0, and no additional normalization of the data was performed. Limma^72^ was used to assess gene enrichment in luminal versus non-luminal samples, and an adjusted p value <0.05 was used as the cutoff for negative or positive enrichment. The correlation of each of the positive and negative regulator hits with *PPARG* expression was evaluated in TCGA Cell 2017 MIBC cohort, excluding samples with *PPARG* amplification and NE-like or stroma-rich subtype as above, using Spearman’s rank correlation coefficients and two-sided p values computed using the R function cor.test. The p values were adjusted using the Benjamini-Hochberg method, and an adjusted two-sided p value of <0.05 was used as the cutoff to identify statistically significant correlations. Gene expression was visualized by heatmap of z-scores, with the rows or columns ordered by Spearman’s correlation for each gene compared to *PPARG*, as indicated in the figure legends. Chromatin immunoprecipitation (ChIP) data and analysis were derived from the Cistrome database and toolkit for Cistrome data browser, respectively.^38^ Pathway enrichment analysis was performed using g:GOST web-based tool provided by the g:Profiler platform^73^ Protein-protein interaction analysis and identification of functional clusters from the CRISPR screen hits was performed using STRING database.^74^

#### RT-qPCR

RNA from cultured cells was purified using the Monarch RNA Cleanup kit (NEB). RNA was first converted into complementary DNA (cDNA) using LunaScript™ RT SuperMix kit (NEB). RT-qPCRs were performed on a ViiA7 instrument (Applied Biosystems) using Luna® Universal qPCR master mix (NEB). Target gene expression was normalized to human *GAPDH*, *RPL32*, and *18S* and analyzed using –ΔΔCt relative quantification method^79^.

#### Flow cytometry

Cells were resuspended in FACS buffer (PBS + 2% FBS + 2.5mM EDTA + 0.05% sodium azide), plus 200 ng/ml propidium iodide (Sigma) or 25 μg/ml 4′,6-diamidino-2-phenylindole (DAPI) (Sigma) to exclude dead cells. Data was acquired on a FACSCanto II with FACSDiva software (BD Biosciences) and data was analyzed using FlowJo analysis software (BD Biosciences).

### Quantification and statistical analysis

Statistical analyses of the TCGA MIBC cohort, including subtype gene enrichment analysis and correlation analysis are describe in detail in the above “bioinformatics analysis” section. Sample number (n) and statistical methods used to assess differences between experimental groups are indicated in each figure legend. Bar graphs are plotted as mean plus or minus standard error of the mean. Statistical significance is denoted by asterisks with ∗p < 0.05, ∗∗p < 0.01, ∗∗∗p < 0.001, and ∗∗∗∗p < 0.0001.

## Data Availability

• Sequencing data from the CRISPR screen have been deposited in the NCBI Sequence Read Archive (SRA) (BioProject ID: PRJNA936268) and are publicly available. • This paper does not report original code. • Any additional information required to reanalyze the data reported in this paper is available from the lead contact upon request.
