# Supplementary material for: A genome-wide CRISPR screen maps endogenous regulators of PPARG gene expression in bladder cancer
Source: iScience. 2023 Mar 30;26(5):106525. doi: 10.1016/j.isci.2023.106525 (PMC10214290; doi:10.1016/j.isci.2023.106525)
Supplement: Document S1. Figures S1–S6 and Tables S1–S4 [file mmc1.pdf]

iScience, Volume *26*

Supplemental information

A genome-wide CRISPR screen maps endogenous regulators of PPARG gene expression in bladder cancer

Davide Tortora, Morgan E. Roberts, Gunjan Kumar, Sudha S. Kotapalli, Elie Ritch, Joshua M. Scurll, Brian McConeghy, Sunita Sinha, Alexander W. Wyatt, Peter C. Black, and Mads Daugaard

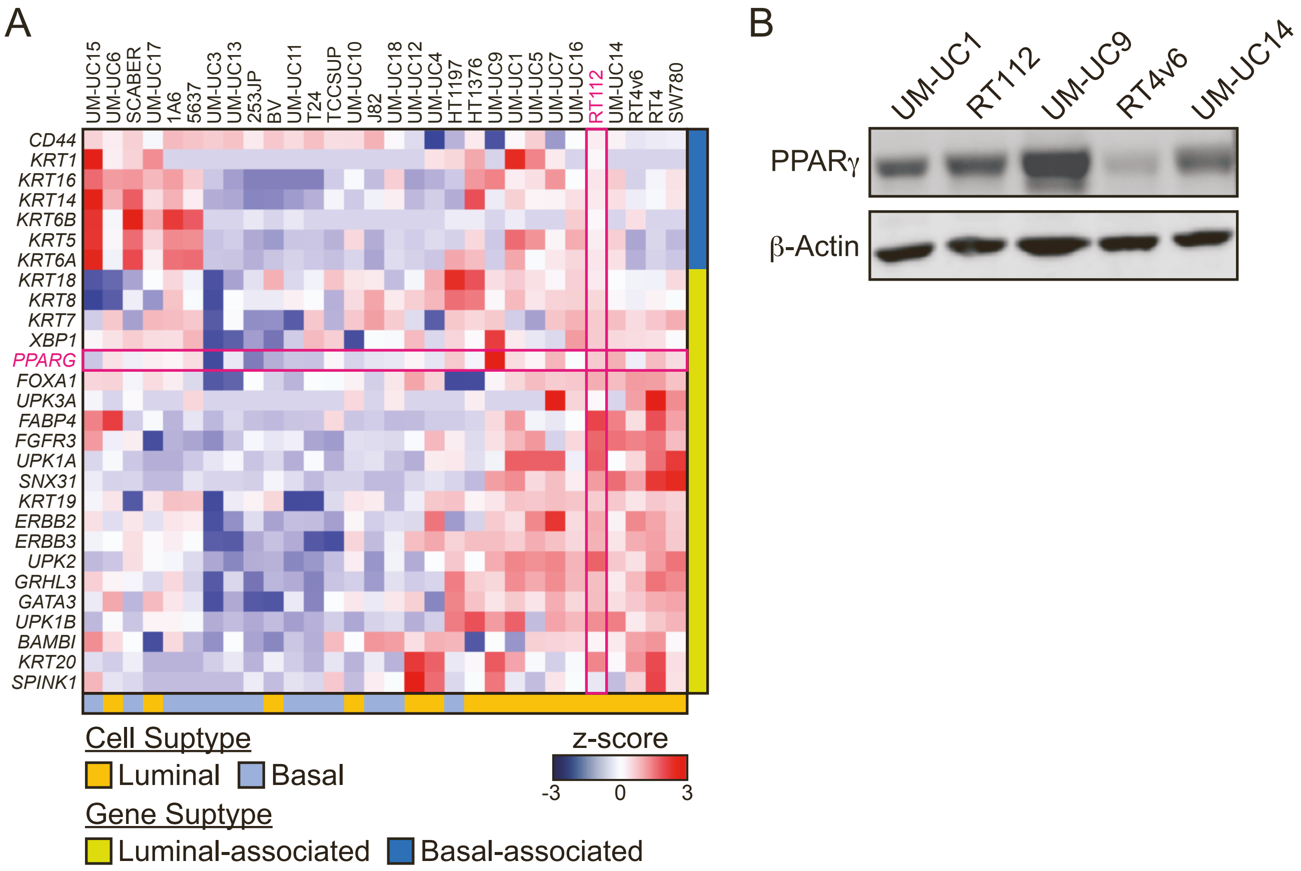

Figure S1. Selection of a luminal bladder cancer cell line for the reporter, related to Figure 1. (**A**) Heat map displaying mRNA expression for genes typical of luminal and basal MIBC, in the indicated bladder cancer cell lines. Expression is represented by z-score. Molecular subtype of each cell line is indicated along the bottom. Molecular subtype associated with each gene is indicated along the right. (**B**) Expression of the indicated proteins in a subset of luminal MIBC cell lines were assessed by Western blot.

A

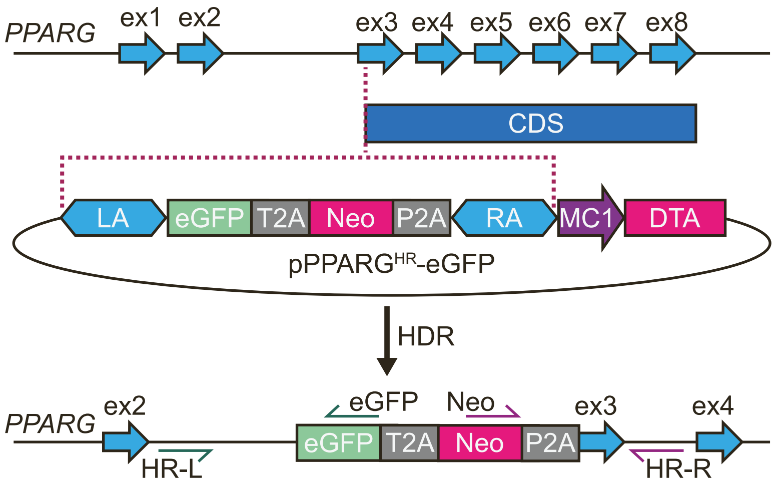

B

Amplicon	WT	PPARγ^{GFP}
HR-L/eGFP	N/A	2719bp
Neo/HR-R	N/A	1311bp

C

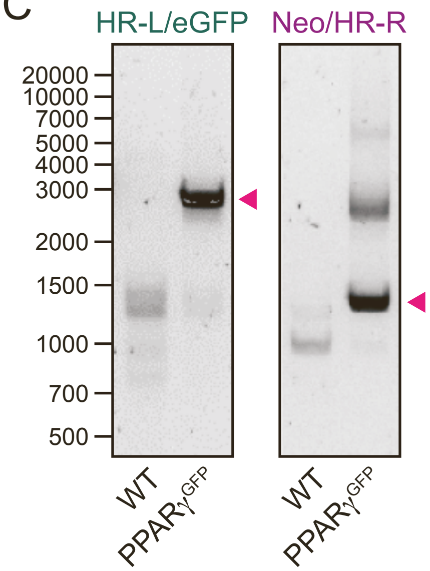

Figure S2. Confirmation of correct site of insertion of the reporter gene, related to Figure 1. (**A**) Diagram illustrating (top) the reporter vector (pPPARG^{HR}-eGFP, 7086bp) and its intended insertion point in the genome (dotted line), and (bottom) the genomic DNA of PPARγ^{GFP} cells following homology directed recombination (HDR). Enhanced green fluorescent protein (eGFP), neomycin resistance gene (Neo), peptide self-cleaving sequences (T2A, P2A), homology arms (LA, RA), MC1 promoter, diphtheria toxin (DTA), validation primers (arrows, HR-L/R eGFP, Neo), and *PPARG* features (coding sequence (CDS), and exons (ex1-8)) are highlighted. (**B**) The expected band sizes (bp) are indicated for each amplicon. (**C**) Agarose gel electrophoresis for the indicated PCR amplicons, which span between the inserted reporter cassette and outside the HDR regions. The pink arrow heads indicate the expected band size.

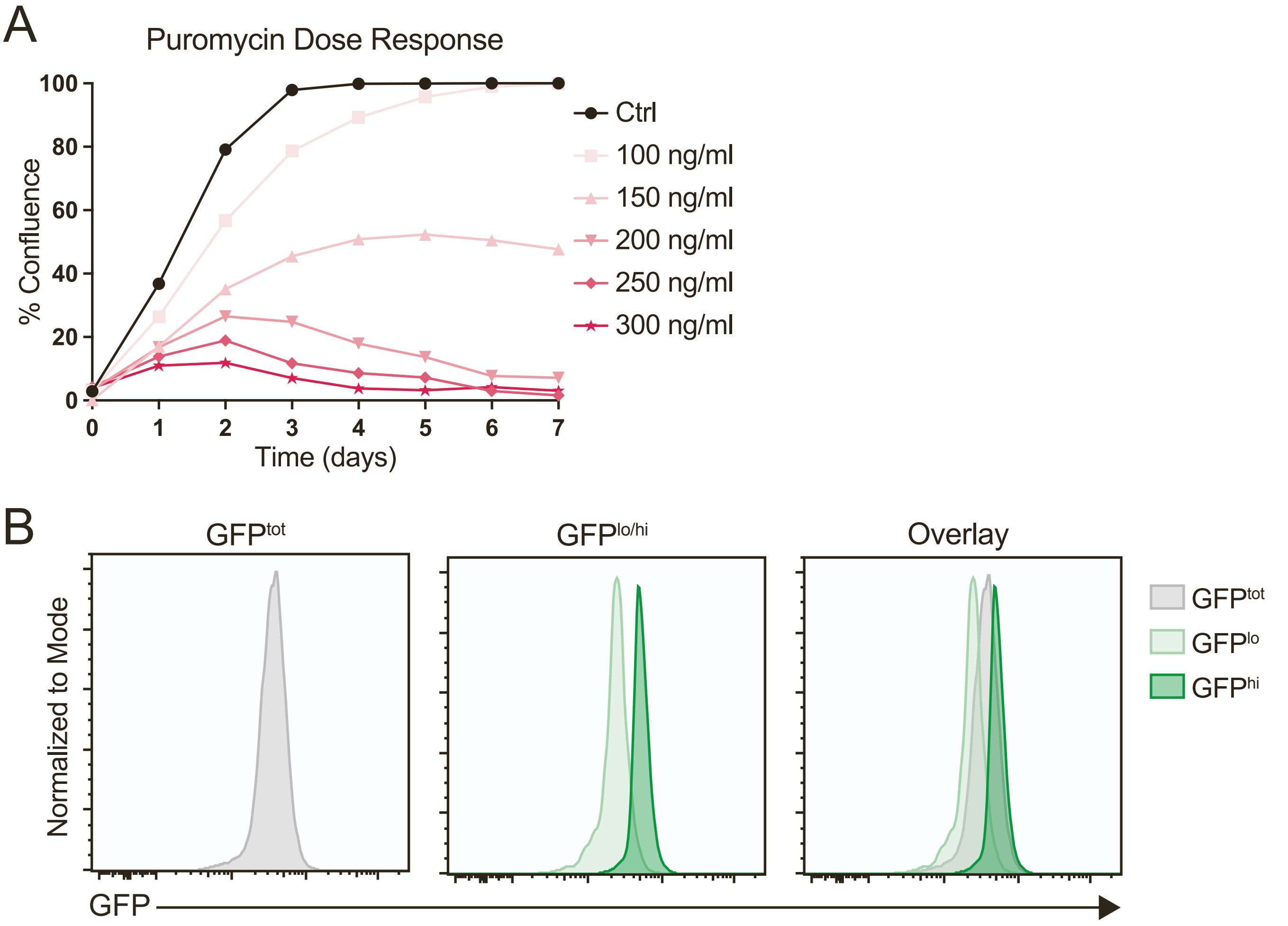

Figure S3. Optimization of the screen parameters and verification of the sorted cells, related to Figure 2. (**A**) Growth curve for RT112-PPARγ^{GFP} under the indicated puromycin concentrations as measured by the Incucyte Live Cell Analysis System. Data represents an average of two wells. (**B**) RT112-PPARγ^{GFP} cells were transduced with a CRISPR lentiviral library and selected for transduced cells followed by sorting for total live cells (GFP^{tot}) or GFP^{lo} and GFP^{hi} cells (based on quartiles). Green fluorescence intensity (eGFP) of the indicated populations was assessed by flow cytometry.

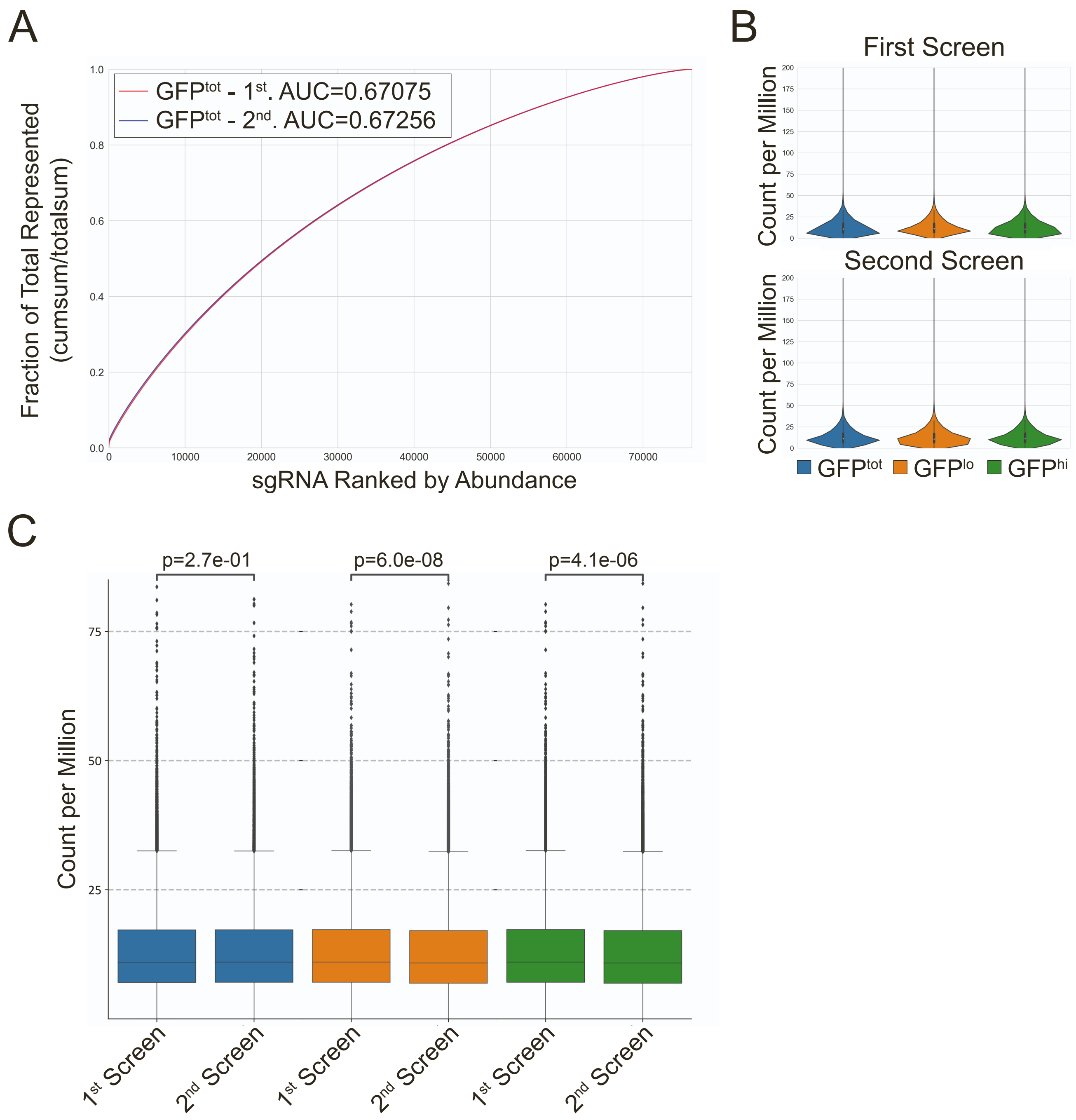

Figure S4. Quality control and comparison of the screen replicates, related to Figure 2. (**A**) Lorenz curve of sgRNA cumulative distribution for GFPtot samples from the two independent experiments. Deviations for the ideal area under the curve (AUC) values are indicated. (**B-C**) The count of each guide present in the screen was normalized as a total per million. (**B**) Distributions of guide representations for each condition in two replicate screens. Each violin is scaled by width and includes a box and whiskers plot at their foci. (**C**) Box-and-whisker plot includes the box showing quartiles. The p-values from Mann-Whitney U test comparing the distributions of guides between replicates 1 and 2 for each sample (GFP^{tot}, GFP^{lo}, GFP^{hi}) are shown.

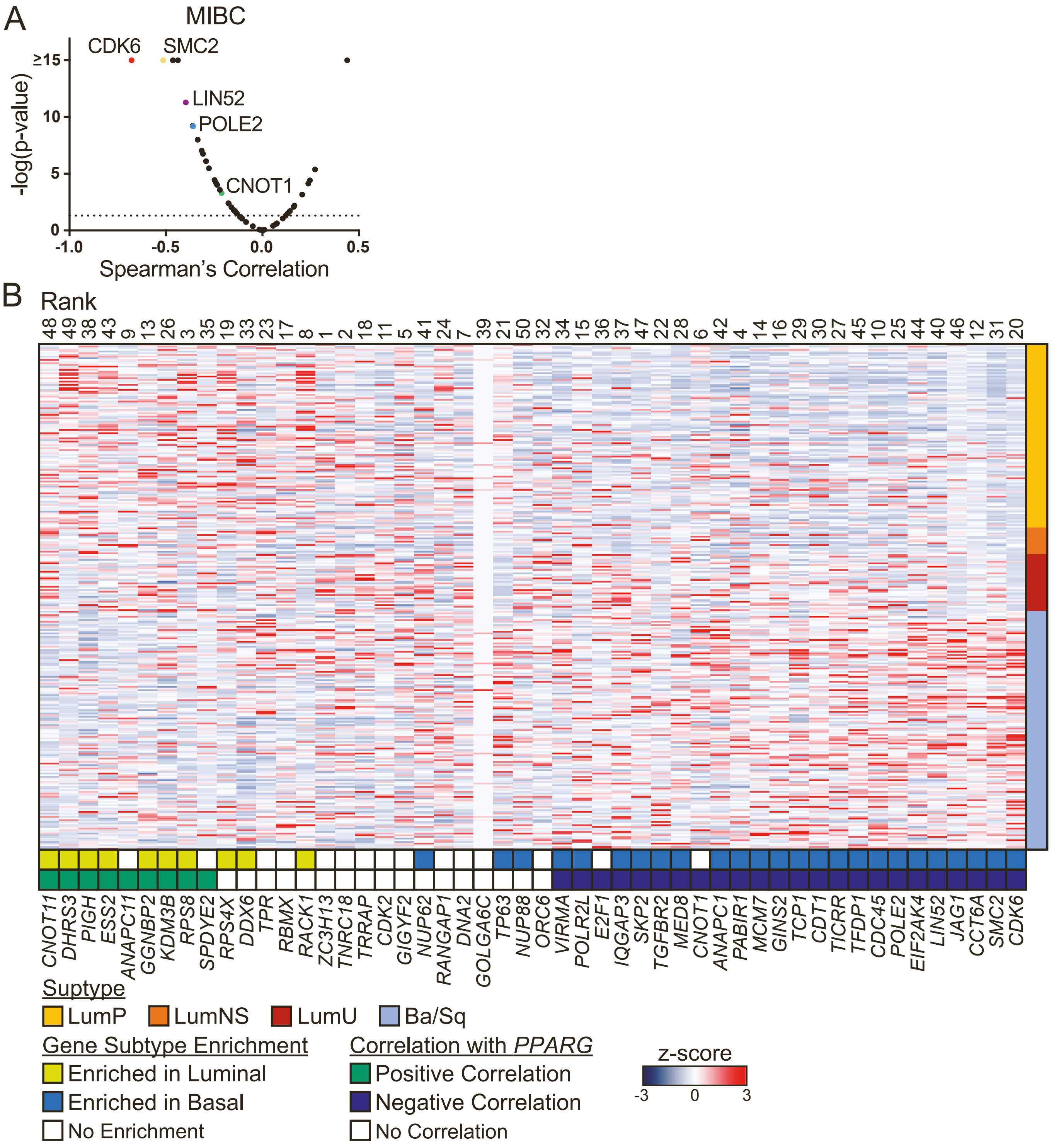

Figure S5. Top negative regulator hits are enriched in non-luminal MIBC, related to Figure 3. The top hits in the GFP^{hi} samples (putative negative regulators) were correlated with *PPARG* gene expression in luminal and basal MIBC tumour samples, excluding samples with *PPARG* copy number amplification (TCGA, n=303) (**A**) Volcano plot represents Spearman's coefficients relative to adjusted p-value for each gene. The dotted line indicates p = 0.05. (**B**) Heatmap displaying mRNA expression (RNAseq V2 RSEM) for each of the hits. Expression is represented by z-score. Columns are ordered from left to right by decreasing Spearman's correlation coefficient based on correlation of each gene with *PPARG*. Adjusted gene rank is indicated on top, and gene IDs are indicated at the bottom. Subtyping of each sample was performed using the MIBC consensus classifier, and are indicated on the right of the plot (LumP = luminal papillary, LumNS = luminal nonspecified, LumU = luminal unstable, Ba/Sq = basal/squamous). Positive (green) and negative (dark blue) correlation of the indicated genes with PPARG (adj. p<0.05) are indicated along the bottom. White boxes indicate no significant correlation. Gene enrichment in luminal (yellow-green; LumP, LumNS, LumU) or basal (blue; Ba/Sq) tumours is indicated along the bottom.

A

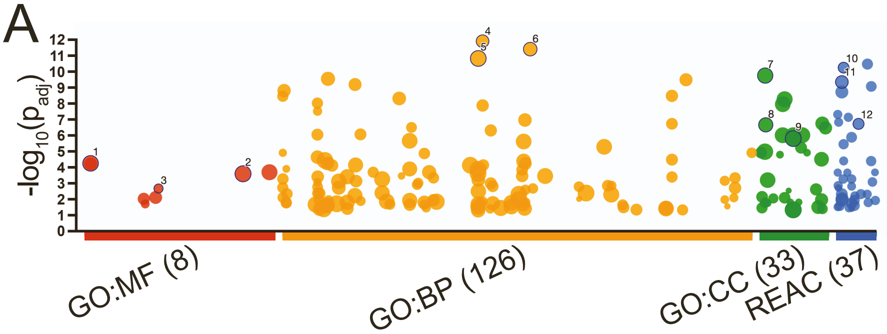

B

#	Term Name	Intersection Size
1	Nucleic acid binding	25
2	Organic cyclic compound binding	27
3	Cyclin binding	3
4	Cell cycle phase transition	17
5	Cellular macromolecule metabolic process	39
6	Regulation of cell cycle	21
7	Nucleoplasm	27
8	Chromosome	17
9	Intracellular non-membrane-bounded organelle	26
10	DNA Replication	12
11	Cell cycle, mitotic	19
12	Mitotic G1 phase and G1/S transition	10

C

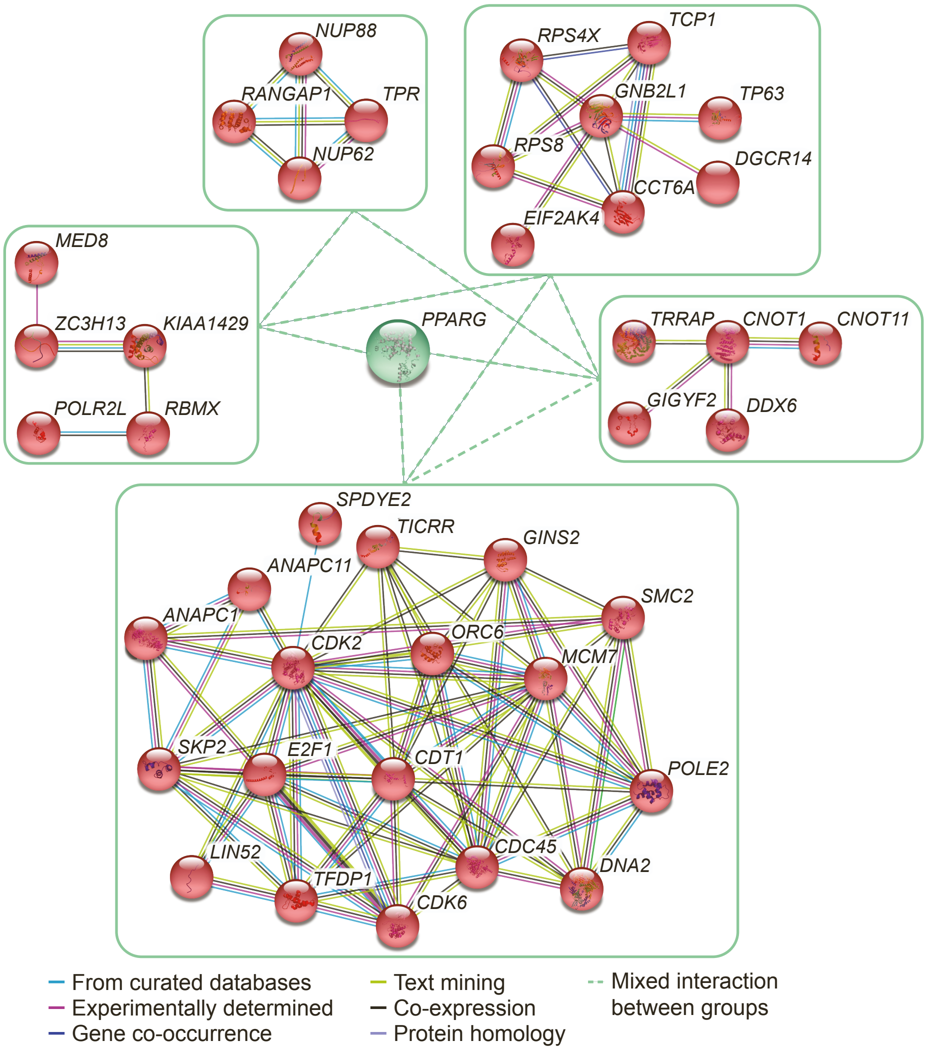

Figure S6. Functional grouping of negative regulator hits, related to Figure 4. (**A-B**) Functional enrichment analysis on the top 50 hits from the GFP^{hi} group was performed with the g:GOSt tool. (**A**) Functional terms (coloured circles) are grouped on the x-axis and colour-coded by data sources (GO = Gene Ontology, REAC = Reactome, BP = biological process, MF = molecular function, CC = cellular component). Bracketed numbers indicate how many significantly enriched terms are from this source. Adjusted enrichment p-values are plotted on the y-axis. The circle sizes are proportional to the corresponding term size. Location of terms is fixed on the x-axis and terms from the same data source are closer to each other. Numbered circles indicate some of the relevant terms, and (**B**) detailed information (term name and number of top hits intersecting with the term) are described in the table. (**C**) Diagram shows interactions between the top hits as determined by the STRING database. Highlighted are the main relationships between some of the top hits, which can be clustered into groups based on function.

Table S1: Putative Negative Regulators, Related to Table 1

Rank	Gene ID	Gene Name
1	ZC3H13	zinc finger CCCH-type containing 13
2	TNRC18	trinucleotide repeat containing 1
3	RPS8	ribosomal protein S8
4	PABIR1	PP2A Aalpha (PPP2R1A) And B55A (PPP2R2A) Interacting Phosphatase Regulator 1
5	GIGYF2	GRB10 interacting GYF protein 2
6	CNOT1	CCR4-NOT transcription complex subunit 1
7	DNA2	DNA replication helicase/nuclease 2
8	RACK1	Receptor for activated C kinase 1
9	ANAPC11	anaphase promoting complex subunit 11
10	CDC45	cell division cycle 45
11	CDK2	cyclin dependent kinase 2
12	CCT6A	chaperonin containing TCP1 subunit 6A
13	GGNBP2	gametogenetin binding protein 2
14	MCM7	minichromosome maintenance complex component 7
15	POLR2L	RNA polymerase II subunit L
16	GINS2	GINS complex subunit 2
17	RBMX	RNA binding motif protein, X-linked
18	TRRAP	transformation/transcription domain associated protein
19	RPS4X	ribosomal protein S4, X-linked
20	CDK6	cyclin dependent kinase 6
21	TP63	tumor protein p63
22	TGFBR2	transforming growth factor beta receptor 2
23	TPR	translocated promoter region, nuclear basket protein
24	RANGAP1	Ran GTPase activating protein 1
25	POLE2	DNA polymerase epsilon 2, accessory subunit
26	KDM3B	lysine demethylase 3B
27	TI CRR	TOPBP1 interacting checkpoint and replication regulator
28	MED8	mediator complex subunit 8
29	TCP1	t-complex 1
30	CDT1	chromatin licensing and DNA replication factor 1
31	SMC2	structural maintenance of chromosomes 2
32	ORC6	origin recognition complex subunit 6
33	DDX6	DEAD-box helicase 6
34	VI RMA	vir Like M6A Methyltransferase Associated
35	SPDYE2	speedy/RINGO cell cycle regulator family member E2
36	E2F1	E2F transcription factor 1
37	IQGAP3	IQ motif containing GTPase activating protein 3
38	PIGH	phosphatidylinositol glycan anchor biosynthesis class H
39	GOLGA6C	golgin A6 family member C
40	LIN52	lin-52 DREAM MuvB core complex component
41	NUP62	nucleoporin 62
42	ANAPC1	anaphase promoting complex subunit 1
43	ESS2	Ess-2 Splicing Factor Homolog
44	EIF2AK4	eukaryotic translation initiation factor 2 alpha kinase 4
45	TFDP1	transcription factor Dp-1
46	JAG1	jagged 1
47	SKP2	S-phase kinase associated protein 2
48	CNOT11	CCR4-NOT transcription complex subunit 11
49	DHRS3	dehydrogenase/reductase 3
50	NUP88	nucleoporin 88

Table S2: Human RT-qPCR Primers, Related to STAR Methods

Primer	Sequence
18S-fw	GCACCACCACCCACGGAATCG
18S-rv	TTGACGGAAGGGCACCACCAG
eGFP-fw	CGTAAACGGCCACAAGTTCA
eGFP-rv	CTTCATGTGGTCGGGGTAGC
GAPDH-fw	ACCACCCTGTTGCTGTAGCCAA
GAPDH-rv	GTCTCCTCTGACTTCAACAGCG
GATA3-fw	GCCCCTCATTAAGCCCAAG
GATA3-rv	TTGTGGTGGTCTGACAGTTCG
PPARG-fw	GGGATCAGCTCCGTGGATCT
PPARG-rv	TGCACTTTGGTACTCTTGAAGTT
PSCA-fw	TGCTGCTTGCCCTGTTGAT
PSCA-rv	CCTGTGAGTCATCCACGCA
RAD21-fw	GGATAAGAAGCTAACCAAAGCCC
RAD21-rv	CTCCCAGTAAGAGATGTCCTGAT
RLP32-fw	CCCCTTGTGAAGCCCAAGA
RLP32-rv	GACTGGTGCCGGATGAACTT
SMC1A-fw	AACCTGCGGGTAAAGACCCT
SMC1A-rv	GGCAAAGGTACGGTCCTCAG
SUPT6H-fw	GGATGAGCAAGGCAACTTGAA
SUPT6H-rv	CACGCCGGTACTTTTGTGCT

Table S3: NGS Primers, Related to STAR Methods

**NGS primers - Forward**	
1	AATGATACGGCGACCACCGAGATCTACACTCTTTCCCTACACGACGCTCTTCCGATCTTAAGTAGAGGCTTTATATATCTTGTGGAAAGGACGAAACACC
2	AATGATACGGCGACCACCGAGATCTACACTCTTTCCCTACACGACGCTCTTCCGATCTATCATGCTTAGCTTTATATATCTTGTGGAAAGGACGAAACACC
3	AATGATACGGCGACCACCGAGATCTACACTCTTTCCCTACACGACGCTCTTCCGATCTGATGCACATCTGCTTTATATATCTTGTGGAAAGGACGAAACACC
4	AATGATACGGCGACCACCGAGATCTACACTCTTTCCCTACACGACGCTCTTCCGATCTCGATTGCTCGACGCTTTATATATCTTGTGGAAAGGACGAAACACC
5	AATGATACGGCGACCACCGAGATCTACACTCTTTCCCTACACGACGCTCTTCCGATCTTCGATAGCAATTCGCTTTATATATCTTGTGGAAAGGACGAAACACC
6	AATGATACGGCGACCACCGAGATCTACACTCTTTCCCTACACGACGCTCTTCCGATCTATCGATAGTTGCTTGCTTTATATATCTTGTGGAAAGGACGAAACACC
7	AATGATACGGCGACCACCGAGATCTACACTCTTTCCCTACACGACGCTCTTCCGATCTGATCGATCCAGTTAGGCTTTATATATCTTGTGGAAAGGACGAAACACC
8	AATGATACGGCGACCACCGAGATCTACACTCTTTCCCTACACGACGCTCTTCCGATCTCGATCGATTTGAGCCTGCTTTATATATCTTGTGGAAAGGACGAAACACC
9	AATGATACGGCGACCACCGAGATCTACACTCTTTCCCTACACGACGCTCTTCCGATCTACGATCGATACACGATCGCTTTATATATCTTGTGGAAAGGACGAAACACC
10	AATGATACGGCGACCACCGAGATCTACACTCTTTCCCTACACGACGCTCTTCCGATCTTACGATCGATGGTCCAGAGCTTTATATATCTTGTGGAAAGGACGAAACACC
**NGS primers – Reverse**	
1	CAAGCAGAAGACGGCATACGAGATTCGCCTTGGTGACTGGAGTTCAGACGTGTGCTCTTCCGATCTCCGACTCGGTGCCACTTTTTCAA
2	CAAGCAGAAGACGGCATACGAGATATAGCGTCGTGACTGGAGTTCAGACGTGTGCTCTTCCGATCTCCGACTCGGTGCCACTTTTTCAA
3	CAAGCAGAAGACGGCATACGAGATGAAGAAGTGTGACTGGAGTTCAGACGTGTGCTCTTCCGATCTCCGACTCGGTGCCACTTTTTCAA
4	CAAGCAGAAGACGGCATACGAGATATTCTAGGGTGACTGGAGTTCAGACGTGTGCTCTTCCGATCTCCGACTCGGTGCCACTTTTTCAA
5	CAAGCAGAAGACGGCATACGAGATCGTTACCAGTGACTGGAGTTCAGACGTGTGCTCTTCCGATCTCCGACTCGGTGCCACTTTTTCAA
6	CAAGCAGAAGACGGCATACGAGATGTCTGATGGTGACTGGAGTTCAGACGTGTGCTCTTCCGATCTCCGACTCGGTGCCACTTTTTCAA

Table S4: siRNA Sequences, Related to STAR Methods

Target Gene-siRNA version	Sequence
AHR-1	GAACAGAGCAUUUACGAAA
AHR-2	GAAAGUGGCAUGAUAGUUU
ARNT-1	GAUCAGAUGUCUAACGAUA
ARNT-2	UCAAGGAGAUCGUUUAUUU
CTCF-1	GAAAGUGGUUGGUAAUAUG
CTCF-2	GAAGAUGCCUGCCACUUAC
GATA3-1	ACUACAAGCUUCACAAUAU
GATA3-2	CGGCAGGACGAGAAAGAGU
MED12-1	GCAGAGAAAUUACGUUGUA
MED12-2	UCACUCAUCUCAUGUUAUA
NIPBL-1	CUGAUAAACUAGAACGAAA
NIPBL-2	GGGAAUAUGAAGAGCGUGA
PPARG	GGAUGCAAGGGUUUCUUCCTT
RAD21-1	GGAAGAAGCAUUUGCAUUG
RAD21-2	GAACAGAGCACCAGCAAUC
RARG-1	GAAAUGACCGGAACAAGAA
RARG-2	UAGAAGAGCUCAUCACCAA
RUNX-1	GACAUCGGCAGAAACUAGA
RUNX-2	CACCGCAAGUCGCCACCUA
SMC1A-1	GCAAUGCCCUUGUCUGUGA
SMC1A-2	CAUCAAAGCUCGUAACUUC
SUPT6H-1	GAACAUGACUUCACAGAUG
SUPT6H-2	CCAGAGACCUUCUACAUUG
